# C-type lectin receptor Dectin3 deficiency balances the accumulation and function of FoxO1-mediated LOX-1^+^ M-MDSCs in relieving lupus-like symptoms

**DOI:** 10.1038/s41419-021-04052-5

**Published:** 2021-09-03

**Authors:** Dan Li, Li Lu, Wei Kong, Xiaoyu Xia, Yuchen Pan, Jingman Li, Jiali Wang, Tingting Wang, Jun Liang, Huan Dou, Yayi Hou

**Affiliations:** 1 The State Key Laboratory of Pharmaceutical Biotechnology, Division of Immunology, Medical School, Nanjing University, Nanjing, PR China; 2 Department of Rheumatology and Immunology, Nanjing Drum Tower Hospital, The Affiliated Hospital of Nanjing University Medical School, Nanjing, PR China; 3 Jiangsu Key Laboratory of Molecular Medicine, Medical School, Nanjing University, Nanjing, PR China

**Keywords:** Cell signalling, Autoimmunity, Cell death and immune response

## Abstract

Recent studies indicate that Toll-like receptors (TLRs) and C-type lectin receptors (CLRs) can function as the signal of pattern recognition receptors, which play a pivotal role in the pathogenesis of the autoimmune disease. Systemic lupus erythematosus (SLE) is a classic autoimmune disease. Previous reports mainly focused on the potential role of TLRs in regulating the development of SLE, but little is known about the role of CLRs in the progression of SLE. Our previous studies showed that the inflammation-mediated accumulation of myeloid-derived suppressor cells (MDSCs) including granulocytic (G-MDSCs) and monocytic (M-MDSCs) participated in the pathogenesis of lupus. Mice deficient in Card9 (the downstream molecule of CLRs) were more susceptible to colitis-associated cancer via promoting the expansion of MDSCs. Whether the abnormal activation of CLRs regulates the expansion of MDSCs to participate in the pathogenesis of lupus remains unknown. In the present study, the expressions of CLRs were examined in both SLE patients and mouse models, revealing the expression of Dectin3 was positively correlated with SLEDAI. Dectin3 deficiency retarded the lupus-like disease by regulating the expansion and function of MDSCs. The mechanistic analysis revealed that Dectin3 deficiency promoted FoxO1-mediated apoptosis of MDSCs. Syk-Akt1-mediated nuclear transfer of FoxO1 increased in Dectin3-deficient MDSCs. Notedly, the accumulation of M-MDSCs mainly decreased in Dectin3^−/−^ lupus mice, and the nuclear transfer of FoxO1 negatively correlated with the expression of LOX-1 on M-MDSCs. The silencing of FoxO1 expression in Dectin3^−/−^ mice promoted the expansion of LOX-1^+^ M-MDSCs in vivo, and LOX-1^+^ M-MDSCs increased the differentiation of Th17 cells. Both LOX-1 expression on M-MDSCs and Dectin3 expression on MDSCs increased in patients with SLE. These data indicated that increased LOX-1^+^ M-MDSCs were related to the exacerbation of SLE development and might be potential target cells for the treatment of SLE.

## Introduction

Previous studies indicated that pattern recognition receptors might participate in the pathogenesis of chronic inflammatory conditions and autoimmune diseases [1]. Toll-like receptors (TLRs) and some members of C-type lectin receptors (CLRs) superfamily play a pivotal role in the autoimmune disease [2–5]. Systemic lupus erythematosus (SLE) is a complex, multi-system autoimmune disease caused by a combination of genetic and environmental factors [6]. Previous reports mainly focused on the potential role of TLRs in regulating the development of SLE [7]; however, recent studies indicated that CLRs played a vital role in the progression of SLE. One study suggested that the defective expression and function of Dectin1 on monocytes contributed to the progression of SLE [2, 4, 5]. Meanwhile, another study indicated that the expression of Dectin1 on dendritic cells from SLE patients increased, enhancing the production of IL-1β and promotes Th17 differentiation [5]. However, the role of CLRs in modulating the innate immune response to participate in the development of SLE remains ambiguous.

Myeloid-derived suppressor cells (MDSCs) are immature heterogeneous myeloid-derived cells characterized by immature state and significant ability to suppress the T-cell response. MDSCs significantly expand during inflammation, tumor, and infection, which are comprised of polymorphonuclear (G-MDSCs) and monocytic (M-MDSCs) [8–10]. G-MDSCs are phenotypically and morphologically similar to neutrophils, and M-MDSCs are more similar to monocytes [9]. Recently, MDSCs were suspected to play a vital role in the pathogenesis of SLE. MDSCs were reported to be increased in peripheral blood of SLE patients and promoted Th17 polarization by secreting Arg-1 in vitro [11]. We previously found that MDSCs promoted IL-1β-mediated Th17 polarization and inhibited Treg differentiation by reactive oxygen species (ROS) production in MRL/lpr mice [12]. Meanwhile, we found that MDSCs induced podocyte injury by increasing ROS in lupus nephritis and mammalian target of rapamycin (mTOR) inhibitor INK128 relieved the symptoms of pristane-induced lupus via downregulating the expansion of MDSCs [13, 14]. The depletion of MDSCs in humanized NOD/SCID mice significantly alleviated the symptoms of SLE [11]. However, the molecular mechanism to regulate the accumulation and function of MDSCs in SLE remains unclear.

The adaptor protein Card9, the downstream signal molecules of CLRs, was reported to attenuate the progression of colitis-associated colon cancer by restricting the expansion of MDSCs [15]. CARD9 also protected against lung cancer development by reducing the expansion of MDSCs and indoleamine 2,3-dioxygenase (IDO) production [16]. The accumulation of fungus-mediated MDSCs was dependent on the activation of Dectin1 [17]. β-Glucan induced the differentiation and function of M-MDSCs via the Dectin1 pathway to enhance antitumor immune response [18]. Recently, some studies implicated some CLRs as risk genes for the progression of autoimmune diseases [19]. However, it remains unknown which CLRs affect MDSCs and involve in the progression of SLE.

This study aimed to investigate the expression of CLRs in the development of SLE. We found that symptoms of lupus were relieved in Dectin3-deficient mice via regulating the accumulation and function of MDSCs. Moreover, Dectin3 promoted the expression of LOX-1 on M-MDSCs to increase the Th17/Treg cell imbalance, indicating that LOX-1^+^ M-MDSCs could be regarded as new target cells for treating lupus.

## Materials and methods

### Mice

Female wild-type (WT) C57BL/6 J mice (6–8 weeks old) were brought from the Model Animal Research Center of Nanjing University (Nanjing, China). Dectin3^−/−^ mice were generated as previously described [20] were crossed five generations onto C57BL/6 J background (96.88%) [15]. WT mice and Dectin3^−/−^ mice were housed under pathogen-free conditions in a 12 h light and dark cycle. All procedures involving mice were based on the institutional guidelines for animal care and approved by the Animal Care Committee at Nanjing University (SCXK-Jiangsu-2019-0056). All animals were acclimatized for 2 weeks before experiments. For establishing the pristane-induced lupus mouse model, the mice were injected with pristane (500 µL) by intraperitoneal injection for the following 7 months. For generating imiquimod-induced mice with lupus, the right ears of the mice were treated with 1.25 mg of 5% imiquimod cream every other day for the following 10 weeks.

### Antibody and reagents

Antibodies against phosphorylated FoxO1 (Ser256, 9641), phosphorylated Akt1 (Ser473, 9018), phosphorylated Syk (Tyr525/526, 2710), FoxO1 (C29H4, 2880s), Akt1 (C73H10, 2938), Syk (D3Z1E, 13198), Bim (2933), Bcl2 (3498), Bax (2772), PCNA (13110), and β-actin (4970) for western blot analysis were bought from Cell Signaling Technology. The antibody against LOX-1 (DF6522) for western blot analysis was obtained from Affinity. The anti-IgG antibody for immunofluorescence staining was bought from Abcam. The enzyme-linked immunosorbent assay (ELISA) kits for mouse dsDNA, total IgG, and IgM were purchased from FMS. The ELISA kits for mouse creatinine and blood urea nitrogen were obtained from Wako Pure Chemical Industries. The ELISA kit for mouse urine protein was procured from Bethyl Laboratories. The anti-CD11b-fluorescein isothiocyanate (FITC) antibody, Gr1-allophycocyanin (APC) antibody, CD4-FITC antibody, CD69-APC antibody, B220-FITC antibody, CD25-APC antibody, CD45-PerCp, Ly6G-phycoerythrin (PE), Ly6C-APC, FoxP3-PE antibody, and IL-17-PE antibody for mouse detection were obtained from Biolegend. The mouse LOX-1-PE-cy7 was bought from the R&D system. The anti-CD45-PerCp antibody, HLA-DR-PE Vio770, CD14-Alexa Fluor488, CD11b-PE-Cy5, CD66b-APC, and CD33-PE for human sample detection were obtained from Biolegend. The human LOX-1-Alexa Fluor 750 and Dectin3-Alexa Fluor 750 were bought from Miltenyi. The human FoxO1-Alexa Fluor 750 was obtained from Novus. The cytokines of GM-CSF and IL-6 were obtained from Miltenyi Biotec. The MDSCs and CD4+ T cells were sorted using magnetic beads bought from Miltenyi Biotec. The purified CD3 and CD28 antibodies were obtained from eBioscience. The probe of carboxyfluorescein succinimidyl ester (CFSE) was brought from Thermo Fisher Scientific. The PI and Annexin-V were obtained from FMS.

### Isolation of MDSCs and MDSC suppression assay

Spleen tissue- and bone marrow (BM)-derived MDSCs in mice were purified using an MDSC isolation kit. CD4+ T cells were pretreated with CFSE following the manufacturer’s protocols. The CD4+ T cells (2 × 10^5^ cells/well) were co-cultured with purified MDSCs at 1:2 ratio in a 96-well round-bottom plate pretreated with 4 μg/mL anti-CD3 monoclonal antibody and 2 μg/mL anti-CD28 monoclonal antibody for 72 h. Then, the cells were collected to detect the proliferation of CD4 +T cells using flow cytometry.

### In vitro generation of mouse MDSCs

The tibiae and femora were removed from 8–10-week-old C57BL/6 J mice, and BM cells were flushed. Then, BM cells were supplemented with Roswell Park Memorial Institute Medium (RPMI) 1640, and 40 ng/mL murine IL-6 and 40 ng/mL granulocyte-macrophage colony-stimulating factor (GM-CSF) were added for 4 days.

### ELISA

Anti-dsDNA, IgG, IgM, BUN, creatinine, and urine proteins were measured using ELISA kits following the manufacturer’s protocols.

### Quantitative reverse transcription-PCR

Total RNA was isolated using TRIzol reagent following the manufacturer’s protocols. Real-time PCR was performed using SYBR green dye on Step One sequence detection system (Applied Biosystems, MA, USA). The relative abundance of genes was calculated using the 2^−ΔΔCT^ method, and GAPDH or PBGD as the internal control. The primers used in the study are listed in Supplementary Table 1.

### Western blot analysis

The cells were lysed with cell lysis buffer (Beyotime P0013B) supplemented with the protease inhibitor complex (Beyotime P1006). The nuclear and cytoplasmic proteins were extracted using a nuclear and cytoplasmic protein extraction kit (Beyotime P0027) following the manufacturer’s protocols. The protein concentrations in the extracts were detected using the BCA assay (Beyotime P0012S). Equal amounts of the protein sample were loaded to sodium dodecyl sulfate–polyacrylamide gel electrophoresis gels. Then, the protein sample was electrotransferred to polyvinylidene difluoride membranes. The membranes were blocked with 5% BSA dissolved in TBST (50 mM Tris/HCl, pH 7.6, 150 mM NaCl, and 0.1% Tween-20) for 2 h at room temperature and incubated with the indicated primary antibody overnight at 4 °C, followed by incubation with appropriate enzyme horseradish peroxidase-linked secondary antibody for 2 h at room temperature. Protein bands were visualized using ECL plus western blotting detection reagents (Millipore, MA, USA). The blot images were captured using the FluorChem8000 imaging system (Alpha-Innotech, CA, USA). The gray values were analyzed using ImageJ gel analysis software [21].

### Histological analysis

The kidneys were fixed with 4% paraformaldehyde in phosphate buffer. Paraffin-embedded samples were sectioned at 3 μm and then stained with hematoxylin and eosin (HE) or periodic acid-Schiff (PAS).

### Immunofluorescence staining

Paraffin-embedded kidney tissues were used in the study. Then, the heat-mediated antigen retrieval was used to treat the slides with citrate buffer. The slides were treated with the primary antibody against mouse IgG-FITC (Servicebio) overnight at 4 °C. The slides were counterstained with DAPI for 5 min. Fluorescence images were captured using a laser scanning confocal microscope (FV3000, Olympus Corporation, Japan).

### Flow cytometry analysis

The BM, kidney, and spleen cells were used to prepare single-cell suspensions. Then, the red cells were lysed, filtered through 70-μm cell strainers, and collected by centrifugation at 300 × *g* for 5 min at 4 °C. After washing, the cells were immediately prepared for flow cytometry. For detecting cell surface markers, the cells were pre-incubated with the primary antibody for 30 min at 4 °C in the dark and then washed with phosphate-buffered saline (PBS). For detecting intracellular markers (IL-17A), the cells were re-stimulated with phorbol myristate acetate (PMA; 25 ng/mL) and ionomycin (250 ng/mL) for 6 h in the presence of brefeldin A prior to the staining of cell surface markers. The Treg cells were surface labeled with CD4 and CD25 for 30 min at 4 °C in the dark, followed by fixation, permeabilization, and intracellular staining with Foxp3 for another 30 min at 4 °C in the dark. The mouse Th17 cells were surface labeled with CD4 for 30 min at 4 °C in the dark, followed by fixation, permeabilization, and intracellular staining with IL-17 for another 30 min at 4 °C in the dark. Flow cytometry was performed on a FACSCalibur flow cytometer (BD Biosciences). The data were analyzed using FlowJo software (Tree Star, OR, USA) [21]. The whole blood was obtained from mice, and peripheral blood mononuclear cells (PBMCs) were isolated following the manufacturer’s protocols for density reagent. The sorting of LOX-1^+^ M-MDSCs and LOX-1^−^ M-MDSCs was performed using BD FACS Aria III.

### Th17 cell and CD4^+^CD25^+^ Foxp3^+^ Treg differentiation

CD4+ T cells were purified from BALB/c mice using a CD4+ T-cell isolation kit. CD4+ cells were stimulated with anti-CD3 (5 μg/mL) and anti-CD28 (5 μg/mL) in the culture with 2.5 ng/mL hTGF-β, 20 ng/mL IL-6, 10 μg/mL anti-IL-4 mAb, and anti-IFN-γ mAb in 24-well plates. In the meantime, pretreated CD11b+ Gr1+ cells were added to the culture on day 0 at a ratio of 1:1, and the cells were cultured in triplicate in the culture medium. On day 3, the cells were stimulated with 5 ng/mL PMA, 1 ng/mL ionomycin, and 10 ng/mL brefeldin A for 5 h. Then cells were stained with FITC-conjugated anti-mouse CD4 mAb. After permeabilization with cytofix/cytoperm, the cells were stained with APC-conjugated anti-mouse IL-17A mAb for 30 min at 4 °C in the dark. After washing with buffer, the cells were analyzed using flow cytometry. After 3-day activation, the supernatants were collected for IL-17A cytokine assays using ELISA following the manufacturer’s protocols. CD4+ T cells were cultured with anti-CD3 (5 μg/mL) and anti-CD28 (5 μg/mL) mAbs in the presence of 5 ng/mL TGF-β in a 24-well plate for 72 h in complete RPMI medium (5 × 10^5^ cells/well). In the meantime, pretreated MDSCs were added to the culture on day 0 at a ratio of 1:1. After 72 h, the cells were permeated with cytofix/cytoperm and then stained with PE-conjugated anti-mouse Foxp3 mAb. After washing, they have stained with FITC-conjugated anti-mouse CD4 mAb and APC-conjugated anti-mouse CD25 mAb for 30 min at 4 °C in the dark. After washing with the buffer, the cells were analyzed using flow cytometry [21].

### Plasmid constructs and transfection

Recombinant vector encoding mouse FoxO1 (NM-019739.3) was created by PCR-based amplification of RAW264.7 cDNA and then subcloned into the pcDNA3.1 eukaryotic expression vector (RiboBio). The constructs were defined by DNA sequencing. The plasmids were transfected into MDSCs with RFectSP Plasmid DNA Transfection Reagent (Changzhou Bio-generating Biotechnology Corp. 21016) following the manufacturer’s protocols. The cells were incubated for 24 h and functionally evaluated using quantitative polymerase chain reaction (qPCR) and western blot analysis.

### siRNA transfection

MDSCs were transfected with FoxO1, Syk, and Akt1 small interfering RNA (siRNA) and NC siRNA and supplemented with RFectSP siRNA/miRNA transfection reagent (Changzhou Bio-generating Biotechnology Corp. 11024) following the manufacturer’s protocols. The cells were incubated for 12 h and functionally evaluated using qPCR and western blot analysis.

### Human samples

A total of 59 patients with SLE who visited the Department of Rheumatology, Nanjing Drum Tower Hospital (Nanjing, China) were prospectively enrolled. All SLE patients were diagnosed according to the criteria set out by American College of Rheumatology revised criteria in 1997 [22]. Patients who had other autoimmune diseases; had a history of familial hyperlipidemia and/or thyroid disease, diabetes mellitus, and/or other rheumatic diseases; and took lipid-lowering agents or thyroid medications were excluded. The disease activity of these patients was measured using the Systemic Lupus Erythematosus Disease Activity Index (SLEDAI) [22]. Disease activity was evaluated using the SLEDAI and a cutoff of ≥8 was used to define active disease (Table 1). This study was approved by the ethics committee at The Affiliated Drum Tower Hospital of Nanjing University Medical School (ID: SC201700201) and undertaken according to the guidelines of the Declaration of Helsinki. At entry, patients completed a standardized medical history, laboratory tests, and analyses. All the detections were carried out at the clinical laboratory of Nanjing Drum Tower Hospital.

**Table 1:** The basic information of SLE patients.

	Active group	Inactive group
	*n* = 35	*n* = 24
Age (year, mean ± SD)	45 ± 8	41.2 ± 5.1
Gender (female/male)	30/5	21/3
SLEDAI < 8		24 (40.6%)
SLEDAI ≥ 8	35 (59.4%)	

### Screening of differentially expressed genes

The samples of MDSCs were analyzed by microarray hybridization in Beijing Oligo Biotech Co. (Beijing, China). The preliminary progression was analyzed after obtaining the raw data. The fold value represented the degree of differential expression between MDSCs of WT mice with lupus and Dectin3-deficient mice with lupus. The standard used to judge differential expression was as follows: the gene expression from the MDSCs of WT mice with lupus was used as a valid gene. Compared with the MDSCs of WT mice with lupus, a fold change of <1.0 indicated a downregulated gene, while a fold change of >1.0 indicated an upregulated gene. Genes with a fold change >1.5 or <0.5 compared with those in the control group were selected for further analysis [23].

### Statistical analysis

The results were expressed as mean ± SEM of three independent experiments, and each experiment included triplicate sets. The data were statistically evaluated using one-way analysis of variances (ANOVA) followed by Dunnett’s test in the control group and multiple-dose groups. A *P* value of ≤ 0.05 indicated a statistically significant difference.

## Results

### Lupus symptoms were relieved in pristane-induced lupus in Dectin3^−/−^ mice

**Fig. 1:**
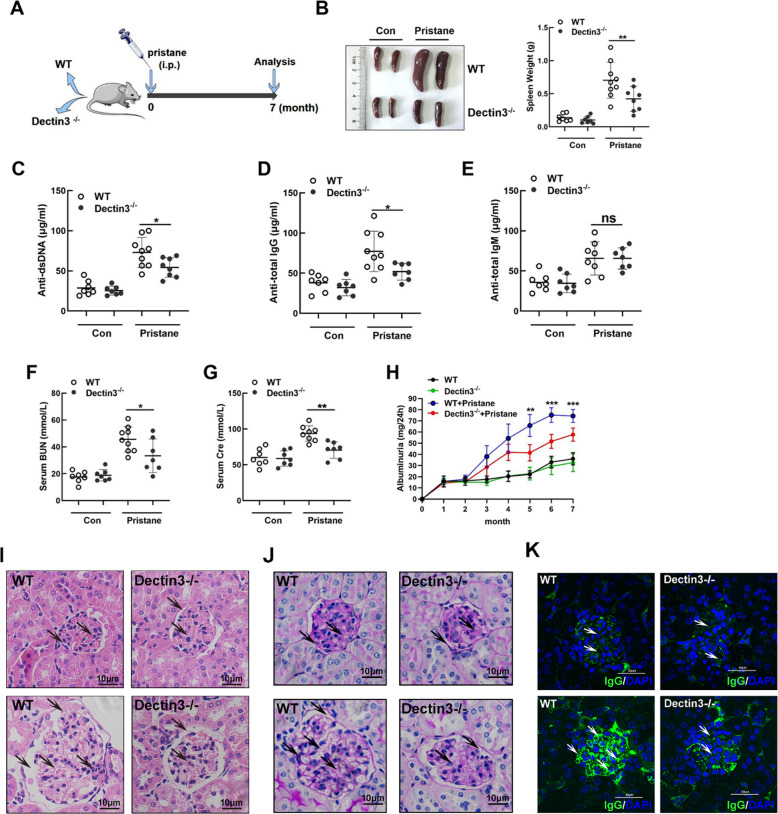
Dectin3 deficiency prevented pristane-induced lupus-like disease. **A** Schematic diagram of a pristine-induced lupus mouse model. **B** Representative photographs of spleens and spleen weights. **C** Serum level of anti-dsDNA was measured using ELISA. **D**, **E** ELISA of the serum levels of total IgG and IgM. **F**, **G** ELISA of the serum levels of BUN and Cre. **H** The level of mouse urine protein was detected using ELISA. **I**, **J** HE and PAS staining of kidney sections (scale bar = 10 μM). **K** IgG deposits in glomeruli were detected by immunofluorescence analysis (scale bar = 30 μM). Data represent the mean scores ± SEM. ^*^P ≤ 0.05, ^*^P ≤ 0.01, ^***^P ≤ 0.001; *n* = 7–9 mice in each group.

We analyzed the expression of CLRs in SLE progression (Fig. S1), suggesting that Dectin3 was a key molecule in the development of SLE patients. To further determine the role of Dectin3 in the lupus process, the pristane-induced lupus model was constructed according to the schematic diagram (Fig. 1A). Splenomegaly reduced in Dectin3^−/−^ lupus mice compared with that in WT lupus mice (Fig. 1B). The serum levels of anti-dsDNA, anti-total IgG, IgM, BUN, and creatinine were reduced in Dectin3^−/−^ lupus mice compared with those in WT lupus mice (Fig. 1C–G). The level of 24 h albuminuria in Dectin3^−/−^ lupus mice was lower than that in WT lupus mice from the fifth month (Fig. 1H). The histopathological renal assessment showed decreased infiltration of crescentic and renal interstitial inflammatory cells in Dectin3^−/−^ mice with lupus compared with WT mice with lupus (Fig. 1I, J). The glomeruli IgG deposits decreased in Dectin3^−/−^ mice with lupus compared with WT mice with lupus, indicating glomerulonephritis with immune complex deposits (Fig. 1K).

The activations of B and T cells of the spleen were inhibited in Dectin3-deficient mice with lupus compared with WT mice with lupus (Figs. S2–3). All the data suggested that Dectin3 deficiency protected against the progression of the lupus-like disease.

### Adoptive transfer of MDSCs from Dectin3^−/−^ mice with lupus relieved imiquimod-induced lupus symptoms

**Fig. 2:**
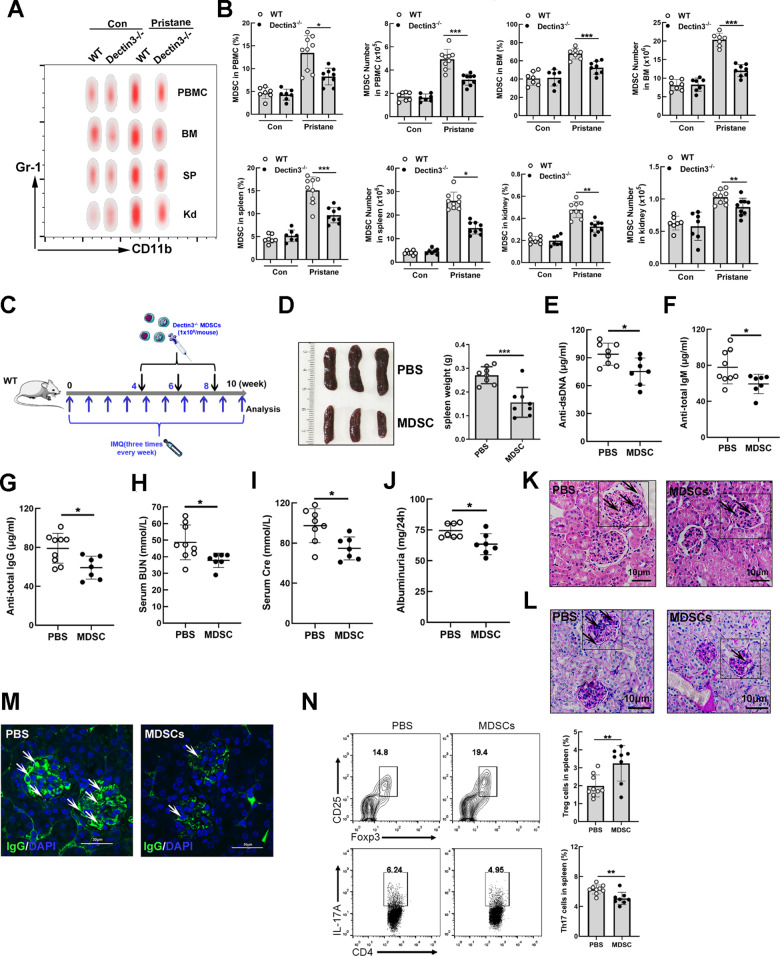
Adoptive transfer of MDSCs from Dectin3^−/−^ mice with lupus alleviated the induction of lupus-like disease. **A** Representative flow cytometry results of MDSCs in PBMC, BM, SP, and Kd of each group. **B** Statistical data of percentages and absolute counts of MDSCs in PBMC, BM, SP, and Kd. **C** Schematic diagram of the adoptive transfer of MDSCs. **D** Representative photographs of the spleen and spleen weights in the MDSC and PBS groups. **E**–**I** ELISA of the serum levels of anti-dsDNA, anti-total IgM, IgG, BUN, and Cre in the MDSC and PBS groups. **J** The level of 24-h mouse urine protein was measured using ELISA. **K**, **L** HE and PAS staining of kidney sections in the MDSC and PBS groups (scale bar = 10 μM). **M** Amounts of IgG deposits in glomeruli were measured using immunofluorescence analysis (scale bar = 30 μM). **N** Flow cytometry analysis detected the percentages of Th17 and Treg cells. Data represent the mean scores ± SEM. ^*^P ≤ 0.05, ^**^P ≤ 0.01, ^***^P ≤ 0.001; *n* = 7–9 mice in each group.

To determine whether Dectin3 promotes the lupus development involved in regulating MDSC accumulation, the change in the numbers of MDSCs was detected by Flow cytometry in PBMCs, BM, SP, and Kd in each group. The percentages and absolute numbers of MDSCs in PBMCs, BM, SP, and Kd were lower in Dectin3^−/−^ mice with lupus than in WT mice with lupus (Fig. 2A, B). Meanwhile, the function of MDSCs was improved in Dectin3^−/−^ mice with pristane-induced lupus (Fig. S4).

Purified MDSCs (1 × 10^6^ cells/mouse) were injected into WT mice with lupus according to the schematic diagram (Fig. 2C). The splenomegaly was relieved in mice with the adoptive transfer of MDSCs (MDSC-group mice) compared with mice in the PBS group (Fig. 2D). The serum levels of anti-dsDNA, anti-total IgM, IgG, BUN, Cre, and albuminuria significantly decreased in the MDSC-group mice compared with the PBS-group mice (Fig. 2E–J). The lupus nephritis was detected by HE and PAS staining and the amount of glomeruli IgG deposits reduced in the MDSC group compared with the PBS group (Fig. 2K–M). The balance of Th17 and Treg cells improved in the MDSC group compared with the PBS group (Fig. 2N). The expansion of MDSCs in PBMC, BM, SP, and Kd significantly reduced in the MDSC group compared with the PBS group (Fig. S5A–H). In addition, the activation of B and T lymphocytes significantly reduced in the MDSC group compared with the PBS group (Fig. S5A–H). These data indicated that Dectin3^−/−^ mice depended mainly on the regulation of MDSCs to relieve lupus progression.

### Dectin3 deficiency promoted FoxO1-mediated apoptosis and reduced the accumulation of MDSCs

**Fig. 3:**
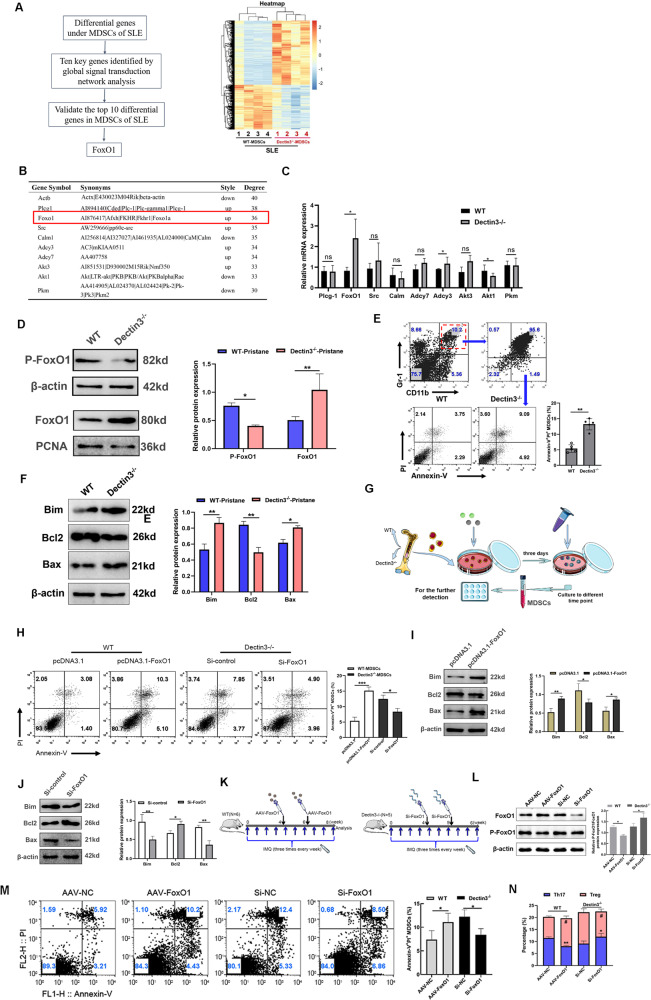
FoxO1 was significantly upregulated in MDSCs of Dectin3^−/−^ mice with lupus and promoted MDSC apoptosis. **A** Analysis pipeline to identify Foxo1 as the downstream markers (left) and gene expression profiling of MDSCs (right). The *mRNA* of MDSCs was extracted and analyzed by gene expression profiling using a Mouse Transcriptome Array (v.1.0) (Affymetrix). **B** Top 10 genes ranked by degree after analysis of signal-net. **C** QPCR validation of microarray data. **D** Phosphorylated FoxO1 and nuclear transfer FoxO1 protein expression were evaluated by western blot analysis in MDSCs isolated from WT mice and Dectin3^−/−^ mice with lupus. **E** Flow cytometry analysis of the apoptosis level of MDSCs from WT and Dectin3^−/−^ mice with lupus. **F** Bim, Bcl2, and Bax protein expression were evaluated by western blot analysis in MDSCs isolated from WT mice and Dectin3^−/−^ mice with lupus. **G** Schematic diagram of BM-MDSC induction. **H** Flow cytometry analysis of the apoptosis level of MDSCs after interfering with si-FoxO1 in Dectin3^−/−^ BM-MDSCs and overexpression with pcDNA3.1-FoxO1 in WT BM-MDSCs. **I**, **J** Bim, Bcl2, and Bax protein expression were detected by western blot analysis in BM-MDSCs after interference or overexpression of FoxO1. **K** Schematic diagram of injection of AAV-FoxO1 and siRNA fragments. **L** P-FoxO1 and FoxO1 protein expression were detected by western blot analysis. **M** Flow cytometry analysis of the apoptosis level of MDSCs. **N** Flow cytometry analysis of Th17 and Treg cell differentiation. Data represent the mean scores ± SEM. ^*^P ≤ 0.05, ^**^P ≤ 0.01, ^***^P ≤ 0.001; *n* = 3–5 in each group.

To explore the molecular mechanism underlying the reduction in MDSC accumulation, MDSCs were isolated from pristane-induced WT and Dectin3^−/−^ mice with lupus to perform transcriptome microarray assays (Fig. 3A). According to the analysis pipeline, we analyzed the differential expression genes of MDSCs from the spleen between WT lupus mice and Dectin3^−/−^ lupus mice, and the results were shown on a heatmap (Fig. 3A). Then, these candidates were addressed by performing a secondary screening by using the global signal-transduction network based on the significantly regulated GOs and pathways to determine the core different genes, and the top 10 genes were found to possess the score of a degree above 30 (Fig. 3B). To further narrow down the candidates, we then used a QPCR assay to screen for the ones that highly correlated with the SLE process in MDSCs isolated from the spleen of WT lupus mice and Dectin3^−/−^ lupus mice. Among these 10 genes, FoxO1, which is involved in regulating cell apoptosis and survival, showed the most change fold in MDSCs from Dectin3^−/−^ lupus mice compared with WT lupus mice, whereas the other candidates showed a little or moderate change (Fig. 3C). In addition, the nuclear transfer of FoxO1 increased in MDSCs of Dectin3^−/−^ mice with lupus (Fig. 3D). The flow cytometry analysis results indicated that the percentage of MDSC apoptosis was higher in Dectin3^−/−^ lupus mice than that in WT lupus mice (Fig. 3E). And the protein expressions of Bim and Bax increased in MDSCs from Dectin3^−/−^ lupus mice compared with WT lupus mice; however, the expression of Bcl2 in MDSCs of Dectin3^−/−^ mice with lupus was lower than that in WT mice with lupus (Fig. 3F). The apoptosis level of MDSCs in Dectin3^−/−^ mice increased following FoxO1 interference (Fig. 3G–J).

To confirm whether Dectin3 promotes FoxO1-mediated MDSC accumulation and abnormal immunomodulatory function in vivo, FoxO1-high-expressed adenovirus (0.2 mL of 1 × 10^11^) and 0.2 mL of empty adenovirus were injected into WT mice with lupus intravenously (Fig. 3K, left). Meanwhile, Si-FoxO1 RNA fragment (0.2 mL of 15 nmol) and 0.2 mL of negative control fragment were injected into Dectin3^−/−^ mice with lupus intravenously (Fig. 3K, right). The results showed that percentages of MDSCs increased in BM, spleen, and kidney of Si-FoxO1 Dectin3^−/−^ mice with lupus compared with Si-NC-group mice (Fig. S6). The nuclear transfer of FoxO1 in Si-FoxO1 Dectin3^−/−^ mice with lupus was lower than that in Si-NC-group mice. The percentage of apoptotic MDSCs decreased in Si-FoxO1 Dectin3^−/−^ mice with lupus compared with Si-NC-group mice (Fig. 3M). These data indicated that Dectin3 deficiency promoted FoxO1-mediated apoptosis to decrease the expansion of MDSCs in lupus development in vitro and in vivo.

### Syk-Akt1-mediated nuclear transfer of FoxO1 reduced in MDSCs of Dectin3^−/−^ mice with lupus

**Fig. 4:**
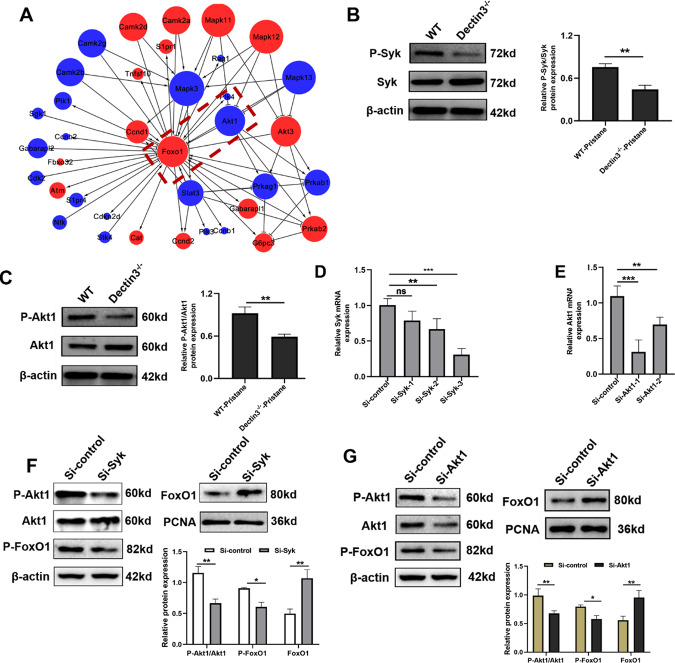
Syk-Akt1-mediated nuclear transfer of FoxO1 was inhibited in Dectin3-deficient MDSCs. **A** Gene co-expression network analysis showed that FoxO1 was significantly related to Akt1. **B** P-Syk and Syk protein expression was evaluated by western blot analysis in MDSCs isolated from WT mice and Dectin3^−/−^ mice with lupus. **C** P-Akt1 and Akt1 protein expression were evaluated by western blot analysis in MDSCs isolated from WT mice and Dectin3^−/−^ mice with lupus. **D** Interference efficiency of RNA fragments of Syk evaluated using qPCR. **E** Interference efficiency of RNA fragments of Akt1 by qPCR. **F** P-Akt1, Akt1, P-FoxO1, and FoxO1 protein expression was detected by western blot analysis in BM-MDSCs after interference with Syk. **G** Western blot detection of P-Akt1, Akt1, P-FoxO1, and FoxO1 protein expression levels after interference with Akt1. Data represent the mean scores ± SEM. ^*^P ≤ 0.05, ^**^P ≤ 0.01, ^***^P ≤ 0.001; *n* = 3 per group.

To explore further the molecular mechanism of regulating the nuclear transfer of FoxO1 in Dectin3-deficient MDSCs, a co-expression network of differentially expressed genes with a hub of FoxO1 was used. FoxO1 is regulated by the PI3K/Akt1 pathway and associated with cell survival and apoptosis progression (Fig. 4A). The phosphorylation of Akt1 and Syk reduced in MDSCs of Dectin3^−/−^ mice with lupus compared with WT mice with lupus (Fig. 4B, C). BM-MDSCs with Syk and Akt1 interference fragments were transfected for 12 and 24 h (Fig. 4D, E) and then treated with R848 for 24 h to confirm whether Dectin3 regulated the nuclear transfer of FoxO1 via the Syk-Akt1 signal. BM-MDSCs transfected with Akt1 RNA fragment significantly reduced the nuclear transfer of FoxO1 after R848 stimulation (Fig. 4F, G). The data showed that Dectin3 inhibited Syk-Akt1-mediated nuclear transfer of FoxO1.

### Interference of FoxO1 promoted the induction of lupus-like disease in Dectin3^−/−^ mice

**Fig. 5:**
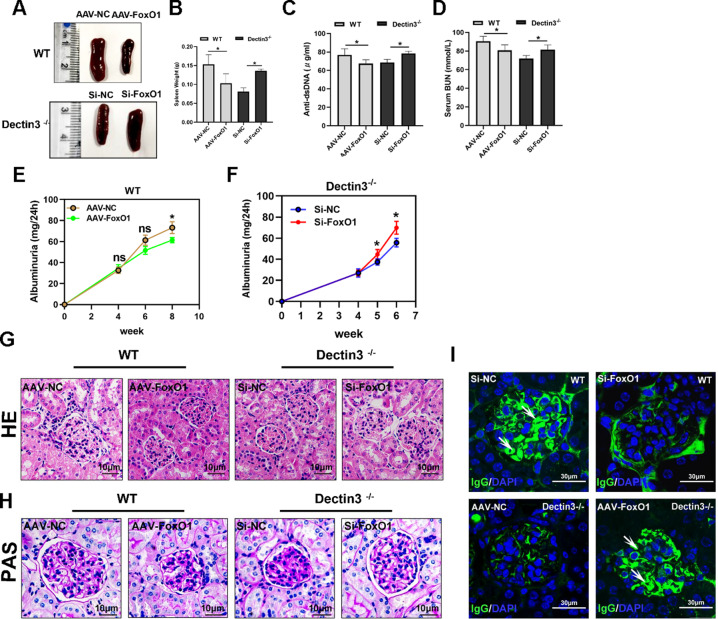
Silencing of FoxO1 expression in Dectin3-deficient mice aggravated the lupus-like disease. **A**, **B** Representative photographs of the spleen and spleen weights. **C**–**F** ELISA analysis of the serum levels of anti-dsDNA, BUN, and 24-h mouse urine protein. **G**, **H** HE and PAS staining of kidney sections (scale bar = 10 μM). **I** Amounts of IgG deposits in glomeruli were measured by immunofluorescence analysis (scale bar = 30 μM). Data represent the mean scores ± SEM. ^*^P ≤ 0.05, ^**^P ≤ 0.01, ^***^P ≤ 0.001; *n* = 5 mice per group.

To further confirm whether Dectin3 promoted lupus progression via inhibiting FoxO1 expression, FoxO1-high-expression adenovirus (0.2 mL of 1 × 10^11^) and 0.2 mL of empty adenovirus were injected into WT mice with lupus intravenously (Fig. 3K, left). Si-FoxO1 RNA fragment (0.2 mL of 15 nmol) and 0.2 mL of negative control fragment were injected into Dectin3^−/−^ mice with lupus intravenously (Fig. 3K, right). The results showed that the splenomegaly in AAV-FoxO1 WT mice with lupus was significantly relieved compared with that in AAV-NC-group mice. However, the splenomegaly in Si-FoxO1 Dectin3^−/−^ mice with lupus was higher than in Si-NC-group mice (Fig. 5A, B). The serum Anti-dsDNA and BUN were higher in Si-FoxO1 Dectin3^−/−^ mice with lupus than in Si-NC-group mice (Fig. 5C, D). The symptoms of lupus nephritis were significantly aggravated in Si-FoxO1 Dectin3^−/−^ mice with lupus compared with Si-NC-group mice, including decreased infiltration of crescentic and renal interstitial inflammatory cells and reduced amounts of glomeruli IgG deposits (Fig. 5E–I). These data indicated that the downregulation of FoxO1 exacerbated the progression of Dectin3^−/−^ lupus-deficient mice.

### FoxO1 negatively regulated LOX-1 expression in M-MDSCs from Dectin3^−/−^ mice

**Fig. 6:**
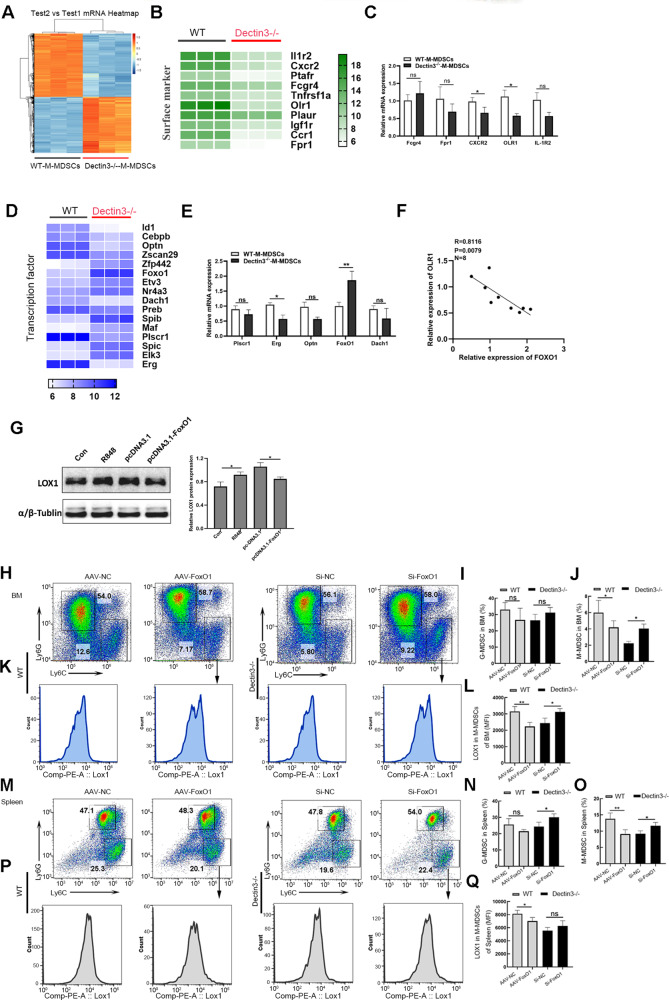
LOX-1 expression negatively correlated with the nuclear transfer of FoxO1 in M-MDSCs. **A** Gene expression profiling of M-MDSCs. The *mRNA* of M-MDSCs was extracted and analyzed by gene expression profiling using a Mouse Transcriptome Array (v.1.0) (Affymetrix). **B** Gene expression profiling of different genes on the surface of M-MDSCs. **C** *mRNA* expression of Fcgr4, Fpr1, CXCR2, IL-1R2, and OLR1 in M-MDSCs of WT mice and Dectin3^−/−^ mice with lupus. **D** Gene expression profiling of differential transcription factors in M-MDSCs. **E** *mRNA* expression of Plscr1, Erg, Optn, Dach1, and FoxO1 in M-MDSCs of WT mice and Dectin3^−/−^ mice with lupus mice. **F** Analysis of the correlation between FoxO1 and OLR1 genes. **G** LOX-1 protein expression was detected by western blot analysis. **H**–**J** Representative flow cytometry results of G-MDSCs and M-MDSCs in each group and the statistical data of percentages of G-MDSCs and M-MDSCs in BM. **K**, **L** Representative flow cytometry results of the LOX-1 expression level in M-MDSCs in the BM of each group. **M**–**O** Representative flow cytometry results of G-MDSCs and M-MDSCs in each group and the statistical data of percentages of G-MDSCs and M-MDSCs in the spleen. **P**, **Q** Representative flow cytometry results of the LOX-1 expression level in M-MDSCs in the spleen of each group. Data represent the mean scores ± SEM. ^*^P ≤ 0.05, ^**^ *P*≤ 0.01, and ^***^P ≤ 0.001; *n* = 3–5 in each group.

Compared with WT lupus mice, the expansion of M-MDSCs significantly decreased in Dectin3^−/−^ lupus mice was revealed (Fig. S7). To perform transcriptome microarray assays so as to explore the molecular mechanism of how Dectin3 influenced M-MDSC, M-MDSCs were isolated from pristane-induced WT mice and Dectin3^−/−^ mice with lupus (Fig. 6A). MDSCs own the heterogeneity, and the differential expression of surface markers on MDSCs has a different role in SLE. Thus, we analyzed different genes of surface markers. The expression of OLR1 (LOX-1 protein gene) significantly decreased in M-MDSCs of Dectin3^−/−^ lupus mice compared with WT lupus mice (Fig. 6B, C). The data suggested that the expression of LOX-1 on M-MDSCs of SP, BM, and Kd significantly decreased in Dectin3^−/−^ lupus mice compared with WT lupus mice (Fig. S8).

To further determine the molecular mechanism of regulating the expression of OLR1 on Dectin3-deficient M-MDSCs, the expression of FoxO1 significantly increased in M-MDSCs of Dectin3^−/−^ mice with lupus compared with WT mice with lupus (Fig. 6D, E). The *mRNA* expression of FoxO1 negatively correlated with OLR1 in Dectin3-deficient M-MDSCs (Fig. 6F). When FoxO1 was further overexpressed in BM-M-MDSCs and then stimulated with R848, the protein expression of LOX-1 increased (Fig. 6G). The expression of LOX-1 on M-MDSCs increased in BM, spleen, and kidney of Si-FoxO1 Dectin3^−/−^ mice with lupus compared with Si-NC-group mice (Fig. 6H–Q) in vivo. Above all, the results suggested that FoxO1 negatively regulated the expression of LOX-1 in M-MDSCs in vitro and in vivo.

### LOX-1^+^ M-MDSCs promoted the differentiation of Th17 cells

**Fig. 7:**
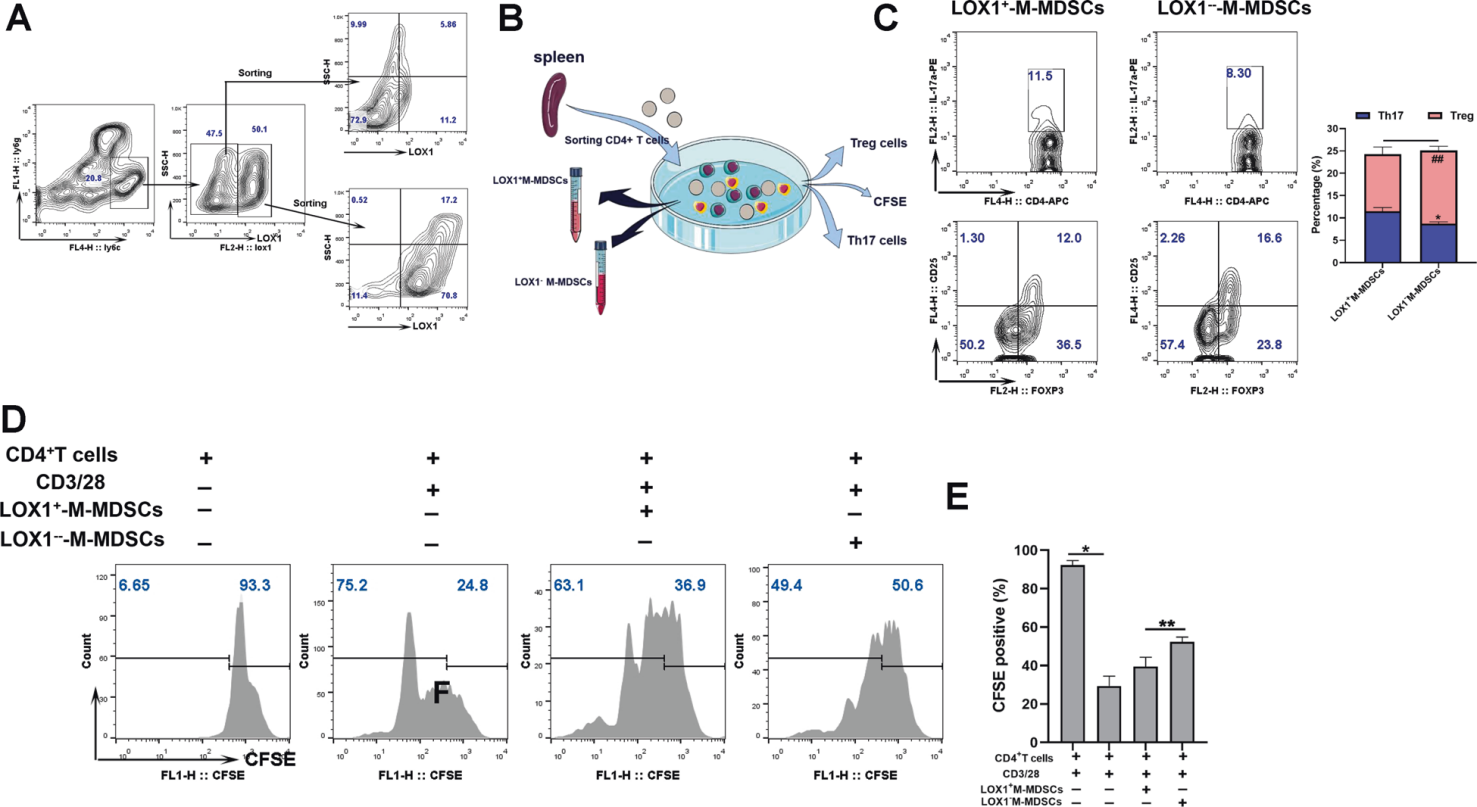
LOX-1^+^ M-MDSCs promoted the differentiation of Th17 cells. **A** Schematic diagram of the sorting of LOX-1^+^ M-MDSCs and LOX-1^−^ M-MDSCs in the spleen. **B** Schematic diagram of co-cultivation of LOX-1^+^ M-MDSCs and LOX-1^−^ M-MDSCs with CD4+ T cells. **C** Flow cytometry analysis of Th17 and Treg cell differentiation. **D**, **E** Flow cytometry analysis of the ability of MDSCs to inhibit T-cell proliferation. Data represent the mean scores ± SEM. ^*^P ≤ 0.05, ^**^P ≤ 0.01, and ^***^P ≤ 0.001; *n* = 3–5 in each group.

To explore the effect of LOX-1 expression on the immunoregulatory function of M-MDSCs. LOX-1^+^ M-MDSCs promoted the accumulation of Th17 cells and inhibited the differentiation of Treg cells, LOX-1^+^ M-MDSCs, and LOX-1^−^ M-MDSCs from mice with lupus were co-cultured with CD4+ T cells from normal mice in different conditional media (Fig. 7A–C). The results showed that the inhibitory effect on the proliferation of CD4+ T cells was lower in LOX-1^+^ M-MDSCs than in LOX-1^−^ M-MDSCs (Fig. 7D). The data indicated that LOX-1^+^ M-MDSCs promoted the accumulation of proinflammatory Th17 cells.

### Increased Dectin3 expression had a positive correlation with the accumulation of LOX-1^+^ M-MDSCs in patients with SLE

**Fig. 8:**
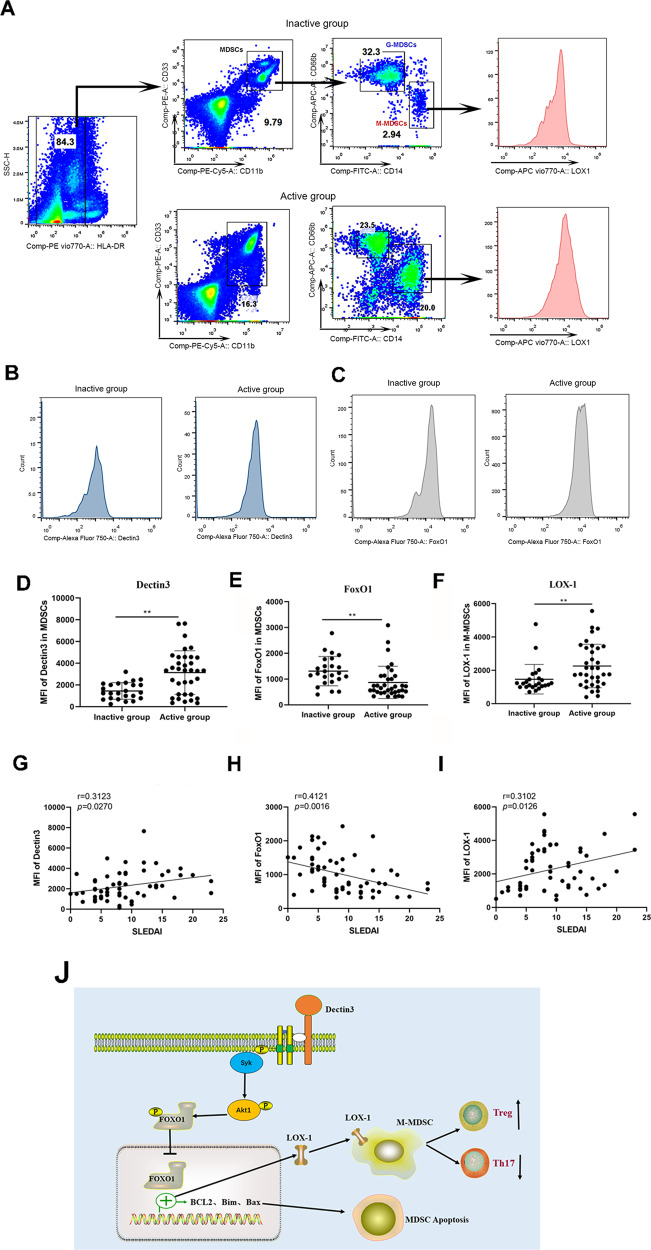
Dectin3 expression positively correlated with the level of LOX-1 on M-MDSCs in patients with SLE. **A** Schematic diagram of flow cytometric detection of peripheral blood MDSCs and M-MDSCs in patients with SLE, and LOX-1 expression detection diagram in M-MDSCs (10 samples in the inactive stage of the disease and 10 samples in the active stage of the disease). **B**, **C** Flow cytometry analysis of Dectin3 (up) and FoxO1 (down) expression in peripheral blood MDSCs. **D**–**F** Statistical data of the expression of Dectin3, FoxO1, and LOX-1 in peripheral blood MDSCs. **G**–**I** The correlations of Dectin3, FoxO1, and LOX-1 expressions with the score of SLEDAI. **J** Schematic diagram of the regulation of the accumulation in Dectin3-deficient mice with lupus. Data represent the mean scores ± SEM. ^*^P ≤ 0.05, ^**^P ≤ 0.01, and ^***^P ≤ 0.001; active group: *n* = 35, inactive group: *n* = 24.

Disease activity was evaluated using the SLEDAI and a cutoff of ≥8 was used to define active disease (Table 1). Flow cytometric analysis of Dectin3 and statistical analysis of relative MFI of Dectin3 on M-MDSCs of peripheral blood from SLE patients (Fig. 8A, B). The results showed that Dectin3 was overexpressed on M-MDSCs of the active SLE group compared with the inactive SLE group (Fig. 8D). Moreover, the expression FoxO1 in M-MDSCs of peripheral blood from SLE patients was detected by flow cytometry (Fig. 8A, C). The downregulation of FoxO1 in M-MDSCs of the active group was detected compared with the inactive group (Fig. 8E). LOX-1 on M-MDSCs of peripheral blood from SLE patients was analyzed by flow cytometry (Fig. 8A) and the results showed that the relative expression of LOX-1 was increased on M-MDSCs of the active SLE group compared with the inactive SLE group (Fig. 8F). Furthermore, the correlation between SLEDAI scores and Dectin3, FoxO1, and LOX-1 levels in M-MDSCs was analyzed by linear regression (stepwise). The data suggested that Dectin3 and LOX-1 levels on M-MDSCs from SLE patients were positively correlated with SLEDAI scores (Fig. 8G, I), whereas the correlation between FoxO1 in M-MDSCs from SLE patients and SLEDAI score was negative (Fig. 8H). All the clinical data indicated that Dectin3 expression probably was positively correlated with LOX-1 level on M-MDSCs in SLE patients, which involved in the FoxO1 pathway.

## Discussion

Dectin3 is mainly expressed on the surface of myeloid cells and is an important type of pattern recognition receptor that can recognize sugar components on the cell wall of pathogens [24, 25]. The role of Dectin3 has been shown to promote antifungal immunity against *Candida spp., Fonsecaea pedrosoi,* and *Blastomyces dermatitidis* infections [18]. Studies reported that Dectin2 might be related to fungal-induced autoimmune diseases [26, 27], and the formation of Dectin3/Dectin2 heterodimer complexes had a greater ability of antifungal immune response. But the role of Dectin3 in autoimmune diseases remains unknown. This study was novel in exploring the role of Dectin3 in the pathogenesis of lupus.

The lupus symptoms of Dectin3^−/−^ mice were relieved compared with those in WT lupus mice (Figs. 1–2). But the mechanism of Dectin3 to regulate the SLE progress remains uncertain. Our previous studies suggested that MDSCs played a vital role in the pathogenesis of SLE [24–26, 28]. However, our previous study indicated Card9 (the downstream adaptor protein of Dectin3) reduced the incidence of colorectal cancer by inhibiting MDSCs recruitment [15]. Meanwhile, Card9 relieved the incidence of lung cancer by reducing IDO production in MDSCs [16]. Whether the role of Dectin3 in regulating the lupus process depends on MDSCs remains unknown.

Therefore, adoptive transfer experiments were addressed to explore whether Dectin3 regulates the lupus process via MDSCs. The data indicated that the spleens of the serum levels of dsDNA antibody, total IgG, and IgM antibody of Dectin3^−/−^ MDSC transplantation group were significantly lower than those in the PBS group (Fig. 2C–J). In addition, the kidney damage was significantly relieved in the MDSC transplantation group compared with the PBS group (Fig. 2K–M). The data indicated that the role of Dectin3 in alleviating lupus symptoms via regulating MDSCs.

To further determine the molecular mechanism of Dectin3 in regulating MDSCs accumulation and function, we analyzed the *mRNA* expression profile of MDSCs in WT and Dectin3^−/−^ lupus mice. The results of the transduction network of the differential expressed genes were analyzed by bioinformatics, combined with the GO and pathway analysis, which revealed that FoxO1 expressed in MDSCs of Dectin3^−/−^ lupus mice lower than that in WT lupus mice.

In addition, studies showed that FoxO1 was mainly involved in pathophysiological processes such as cell proliferation, apoptosis, and oxidative stress [29–32]. Researches showed that FoxO1 *mRNA* expression in PBMCs of SLE patients was lower than that in healthy controls, which negatively correlated with SLEDAI. Our results indicated that Dectin3 negatively correlated with FoxO1 gene expression in MDSCs isolated from lupus mice.

The bioinformatics analysis showed that the FoxO1 signal was mainly concentrated in the biological processes of MDSC apoptosis, oxidative stress, cell proliferation, and so forth. In MDSCs of Dectin3^−/−^ mice with lupus, the phosphorylation level of FoxO1 was significantly downregulated, and the level of nuclear metastasis of FoxO1 significantly increased (Fig. 3C). At the same time, the expression levels of pro-apoptotic proteins Bim and Bax in MDSCs of Dectin3^−/−^ mice with lupus significantly increased, and the expression of anti-apoptotic protein Bcl2 significantly decreased (Fig. 3D). The aforementioned results indicated that Dectin3 regulated the number of MDSCs by regulating the nuclear transfer level of FoxO1. FoxO1 (Thr24, Ser256, and Ser319) was phosphorylated by Akt, which was transported from the nucleus to the cytoplasm, resulting in the loss of transcriptional activity, thereby inhibiting the expression of downstream genes regulated by FoxO1. The acetylation of FoxO1 weakened the ability to bind to homologous DNA sequences while enhancing the phosphorylation of FoxO1, further reducing its transcriptional activity [29–31, 33, 34].

Dectin3 can induce Card9/Bcl-10/Malt1 to form a complex by activating Syk, which in turn activates NF-κB and other pathways, initiating the innate immune response, and mediates adaptive immune response [32, 35]. In addition, studies pointed out that the Syk gene could bind to the promoter region of Akt1 to promote its transcriptional activation, thereby promoting cell proliferation [36, 37]. Dectin3 inhibited the nuclear metastasis of FoxO1 in lupus MDSCs through the Syk-Akt1 signal axis and participated in the development of lupus.

Recent studies showed that a large number of accumulated M-MDSCs were involved in the development of lupus [38]. The exact mechanism of the increase in the number of MDSCs in patients and mice with lupus is still unclear. This may be related to the lack of specific surface markers of MDSCs, which, to some extent, hinders the understanding of the complex regulatory process of MDSCs. The magnetic bead sorting method was used to sort and purify the M-MDSCs in the spleen of WT mice and Dectin3^−/−^ mice with lupus followed by gene chip analysis.

Studies confirmed that G-MDSCs with the high expression of LOX-1 in the tumor environment had a stronger immunosuppressive function on T cells and promoted tumorigenesis [39–41]. Also, low-density granulocytes with high LOX-1 expression in the lupus environment had no significant immunosuppressive ability on T cells, but promoted the production of inflammatory T cells and the development of lupus [42]. LOX-1^+^ M-MDSCs in the lupus environment inhibited the proliferation of T cells to a lesser extent compared with LOX-1^−^ M-MDSCs and promoted Th17/Treg imbalance.

The M-MDSCs in the spleens of different groups of mice with lupus were further sorted and enriched, revealing that the apoptosis level of M-MDSCs after knocking down FoxO1 significantly decreased, and the increase in the number of inflammatory Th17 cells was promoted in Dectin3^−/−^ lupus mice. These data indicated that LOX-1^+^ M-MDSCs were related to the exacerbation of the lupus process and might be potential target cells for lupus.

## Supplementary information

Supplementary Figure legend

Supplementary Tables

Supplementary Figure.1

Supplementary Figure.2

Supplementary Figure.3

Supplementary Figure.4

Supplementary Figure.5

Supplementary Figure.6

Supplementary Figure.7

Supplementary Figure.8

## Data Availability

All data generated or analyzed during the study are included in the article.
