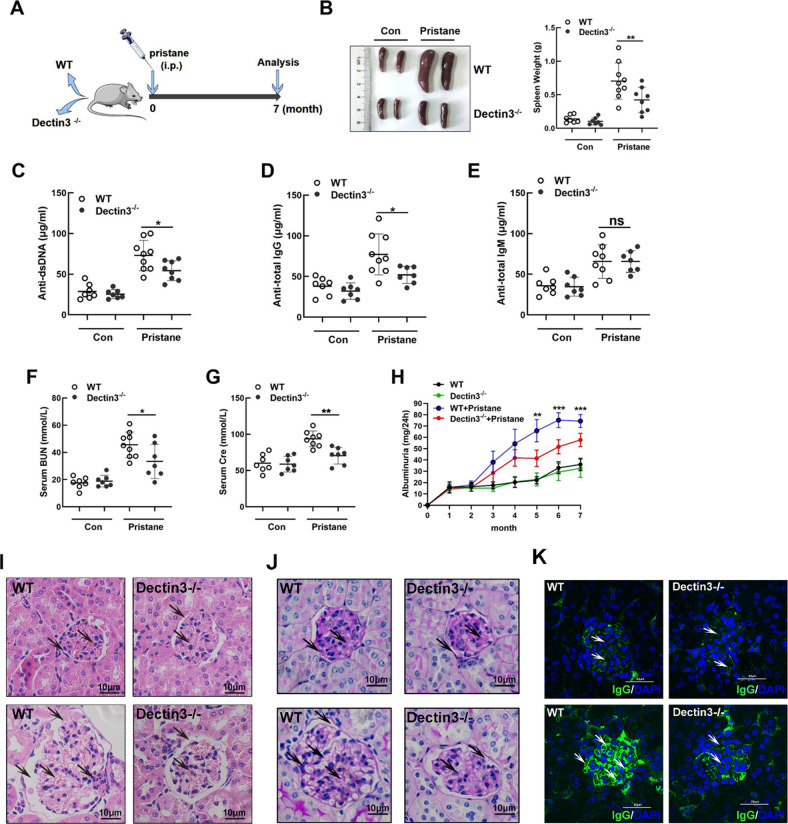# Correction: C-type lectin receptor Dectin3 deficiency balances the accumulation and function of FoxO1-mediated LOX-1^+^ M-MDSCs in relieving lupus-like symptoms

**DOI:** 10.1038/s41419-023-05809-w

**Published:** 2023-04-25

**Authors:** Dan Li, Li Lu, Wei Kong, Xiaoyu Xia, Yuchen Pan, Jingman Li, Jiali Wang, Tingting Wang, Jun Liang, Huan Dou, Yayi Hou

**Affiliations:** 1 The State Key Laboratory of Pharmaceutical Biotechnology, Division of Immunology, Medical School, Nanjing University, Nanjing, PR China; 2 Department of Rheumatology and Immunology, Nanjing Drum Tower Hospital, The Affiliated Hospital of Nanjing University Medical School, Nanjing, PR China; 3 Jiangsu Key Laboratory of Molecular Medicine, Medical School, Nanjing University, Nanjing, PR China

**Keywords:** Cell signalling, Autoimmunity, Cell death and immune response

Correction to: *Cell Death and Disease* 10.1038/s41419-021-04052-5, published online 03 September 2021

The original version of this article contained a figure error. The authors state the following: when we carefully read the published papers again, we realized that we mistakenly put Fig. 2C-M in the position of Figure 1. We provide the right Figure 1 to replace the wrong “Figure 1” as shown below. The original article has been corrected.