# Supplementary material for: C-type lectin receptor Dectin3 deficiency balances the accumulation and function of FoxO1-mediated LOX-1+ M-MDSCs in relieving lupus-like symptoms
Source: Cell Death Dis. 2021 Sep 3;12(9):829. doi: 10.1038/s41419-021-04052-5 (PMC8417277; doi:10.1038/s41419-021-04052-5)
Supplement: Supplementary file 1 — Supplementary Figure legend [file 41419_2021_4052_MOESM1_ESM.docx]

**Figure S1. C-type lectin receptor Dectin3 was significantly increased in lupus mice and SLE patients. Related to Figure 1.** (A-D) The mRNA expressions of Dectin1, Dectin2, Dectin3 and mincle in PBMC, SP, BM and Kd in each group (n=5-7). (E) The mRNA expressions of Dectin1, Dectin2, Dectin3 and mincle in blood of SLE patients (n=10-14). (F) The correlation of Dectin3 mRNA expression with the score of SLEDAI. (G) The correlation of mincle mRNA expression with the score of SLEDAI. Data represent the mean scores ± SEM. *P≤0.05, **P≤0.01, ***P ≤0.001.

**Figure S2. Dectin3 deficiency alleviates the activation of B and T cells and improves the balance of Th17 and Treg cells in pristane-induced lupus mice. Related to Figure 1.** (A) Representative flow cytometry results and the statistics data of percentage of activated T cells in spleen. (B) Representative flow cytometry results and the statistics data of percentage of activated B cells in spleen. (C) Representative flow cytometry results and the statistics data of percentage of Treg cells in spleen. (D) Representative flow cytometry results and the statistics data of percentage of Th17 cells in spleen. Data represent the mean scores ± SEM. *P≤0.05, **P≤0.01, ***P ≤0.001. n=7-9 mice per group.

**Figure S3. Dectin3 deficiency prevents imiquimod-induced lupus-like disease. Related to Figure 1.** (A) Schematic diagram of imiquimod-induced lupus mouse model. (B) Representative photographs of spleens and spleen weights. (C-G) The serum levels of Anti-dsDNA, total IgG, IgM, BUN and Cre were measured by ELISA. (H) The level of mouse urine protein was detected by ELISA. (I-J) HE and PAS staining of kidney sections (scale bar=10μM). Data represent the mean scores ± SEM. *P≤0.05, **P≤0.01, ***P ≤0.001. n=7-8 mice per group.

**Figure S4. Dectin3 deficiency improves the function of MDSCs in pristane-induced lupus mice. Related to Figure 2.** (A) The mRNA expressions of TLR7, Arg-1, IL-10, IL-1β and IDO in MDSCs isolated from WT and Dectin3 lupus mice were analyzed by QPCR. (B) Flow cytometry analysis the ability of MDSCs to inhibit T cell proliferation. (n=5 per group). (C) Schematic diagram of co-cultivation of MDSCs and CD4+ T cells. (D) Flow cytometry analysis detected Th17 and Treg cells differentiation (n=3 per group). Data represent the mean scores ± SEM. *P≤0.05, **P≤0.01, ***P ≤0.001. n=7-9 mice per group.

**Figure S5.** **The expansion of MDSCs in WT lupus mice is reduced after adoptive transfer of MDSCs from Dectin3^-/-^ lupus mice. Related to Figure 2.** (A) Representative flow cytometry results of MDSCs in PBMC of MDSCs group and PBS group. (B) The statistics data of percentage and absolute count of MDSCs in PBMC. (C) Representative flow cytometry results of MDSCs in BM of MDSCs group and PBS group. (D) The statistics data of percentage and absolute count of MDSCs in BM. (E) Representative flow cytometry results of MDSCs in spleen of MDSCs group and PBS group. (F) The statistics data of percentage and absolute count of MDSCs in spleen. (G) Representative flow cytometry results of MDSCs in kidney of MDSCs group and PBS group. (H) The statistics data of percentage and absolute count of MDSCs in kidney. Data represent the mean scores ± SEM. *P≤0.05, **P≤0.01, ***P ≤0.001. n=7-9 mice per group.

**Figure S6. Interference of FoxO1 in vivo aggravates the accumulation of MDSCs in Dectin3-deficient lupus mice. Related to Figure 3.** (A) Representative flow cytometry results of MDSCs in BM of MDSCs in each group. (B) The statistics data of percentage of MDSCs in BM. (C) Representative flow cytometry results of MDSCs in spleen of MDSCs in each group. (D) The statistics data of percentage and absolute count of MDSCs in spleen. (E) Representative flow cytometry results of MDSCs in kidney of MDSCs in each group. (F) The statistics data of percentage and absolute count of MDSCs in kidney. Data represent the mean scores ± SEM. *P≤0.05, **P≤0.01, ***P ≤0.001. n=5 mice per group.

**Figure S7. The expansions of M-MDSCs are reduced in Dectin3-deficent lupus mice. Related to Figure 6.** (A) Representative flow cytometry results of G-MDSCs and M-MDSCs in PBMC. (B-C) The statistics data of percentage and absolute count of G-MDSCs and M-MDSCs in PBMC. (D) Representative flow cytometry results of G-MDSCs and M-MDSCs in BM. (E-F) The statistics data of percentage and absolute count of G-MDSCs and M-MDSCs in BM. (G) Representative flow cytometry results of G-MDSCs and M-MDSCs in spleen. (H-I) The statistics data of percentage and absolute count of G-MDSCs and M-MDSCs in spleen. Data represent the mean scores ± SEM. *P≤0.05, **P≤0.01, ***P ≤0.001. n=7-9 mice per group.

**Figure S8. The expansions of LOX-1+M-MDSCs are reduced in Dectin3-deficent lupus mice.** (A) Representative flow cytometry results of LOX-1 expression on G-MDSCs and M-MDSCs in spleen. (B-C) The statistics data of percentage of LOX-1 expression on G-MDSCs and M-MDSCs in spleen. (D) Representative flow cytometry results of LOX-1 expression on G-MDSCs and M-MDSCs in BM. (E-F) The statistics data of percentage of LOX-1 expression on G-MDSCs and M-MDSCs in BM. (G) Representative flow cytometry results of LOX-1 expression on G-MDSCs and M-MDSCs in kidney. (H-I) The statistics data of percentage of LOX-1 expression on G-MDSCs and M-MDSCs in kidney. Data represent the mean scores ± SEM. *P≤0.05, **P≤0.01, ***P ≤0.001. n=7-9 mice per group.
