# Supplementary material for: C-type lectin receptor Dectin3 deficiency balances the accumulation and function of FoxO1-mediated LOX-1+ M-MDSCs in relieving lupus-like symptoms
Source: Cell Death Dis. 2021 Sep 3;12(9):829. doi: 10.1038/s41419-021-04052-5 (PMC8417277; doi:10.1038/s41419-021-04052-5)
Supplement: Supplementary file 2 — Supplementary Tables [file 41419_2021_4052_MOESM2_ESM.docx]

|  | Sequence |
| --- | --- |
| *FoxO1*  *Syk*  *Akt1*  *GAPDH*  *Plscr1*  *Optn*  *Dach1*  *Erg*  *Fcgr4*  *Fpr1*  *CXCR2*  *OLR1*  *IL-1R2*  *PBGD* | Forward: CCCAGGCCGGAGTTTAACC  Reverse: GTTGCTCATAAAGTCGGTGCT  Forward: CTACCTGCTACGCCAGAGC  Reverse: GCCATTAAGTTCCCTCTCGATG  Forward: ATGAACGACGTAGCCATTGTG  Reverse: TTGTAGCCAATAAAGGTGCCAT  Forward: AGGTCGGTGTGAACGGATTTG  Reverse: TGTAGACCATGTAGTTGAGGTCA  Forward: GGTATCCCCCTCCGTATCCAC  Reverse: GCCACCACCTGCATAACCT  Forward: ATGTCCCATCAACCTCTGAGC  Reverse: TCAAATCGCCCTTTCATAGCTTG  Forward: CCTGGGAAACCCGTGTACTC  Reverse: AGATCCACCATTTTGCACTCATT  Forward: CCAGCAGCTCATATTAAGGAGG  Reverse: GCACACTCAAATAGTGACTGGTC  Forward: ATGTGGCAGCTACTACTACCA  Reverse: ACCCACTTGGGGTCTAGGTTC  Forward: CATTTGGTTGGTTCATGTGCAA  Reverse: CATTTGGTTGGTTCATGTGCAA  Forward: ATGCCCTCTATTCTGCCAGAT  Reverse: GTGCTCCGGTTGTATAAGATGAC  Forward: CAAGATGAAGCCTGCGAATGA  Reverse: ACCTGGCGTAATTGTGTCCAC  Forward: GTTTCTGCTTTCACCACTCCA  Reverse: GAGTCCAATTTACTCCAGGTCAG  Forward: CTGCAAGCGGGAAAACCCT |
|  | Reverse: CTCCAGATGCGGGAACTTTCT |

**Supplementary Table1. Primers for PCR.**

**Supplementary Table2. Sequence of small interfering RNA fragment.**

| Gene | Sequence |
| --- | --- |
| si-FoxO1-1  si-FoxO1-2 | Target gene: GCACCGACTTTATGAGCAA  Target gene: CAGCAACGATGACTTTGAT |
| si-FoxO1-3  si-Syk-1 | Target gene: GGGAGAATGTTCGCTTTCT   \| Sense5’UUAAGU UCCCUC UCGAUGGTG 3’ \| \| --- \| \| Antisense5’ UUCCAUUGGUCUUUGACCCTA 3’ \| |
| si-Syk-2 | \| Sense5’ GGCAGCUAGUGGAACAUUATT 3’ \| \| --- \| \| Antisense5’ UAA UGU UCC ACU AGC UGCCAG 3’ \| |
| si-Syk-3 | \| Sense5’ CCAUCGAGAGGGAACUUAATT 3’ \| \| --- \| \| Antisense5’ UUA AGU UCC CUC UCGAUGGTG 3’ \| |
| si-Akt1-1 | Sense5’CCGGTGCTACTTCCTCCTCAAGAACGCTCGAGCGTTC TTGAGGAAGAGCTTTTTG 3’ |
| si-Akt1-2 | Antisense5’ AATTCAAAAAGCTACTTCCTCCTCAAGAACGCTCG  AGCGTTCTTGAGGAGGAAGTAGCA3’  Sense5’ CCGGTGGACGGGCACATCAAGATAACCTCGAGGTTAT  CTTGATGTGCCCGTCCTTTTTG3’  Antisense5’ AATTCAAAAAGGACGGGCACATCAAGATAACCTC  GAGGTTATCTTGATGTGCCCGTCCA3’ |
|  |  |
