# Supplementary figures and images for: C-type lectin receptor Dectin3 deficiency balances the accumulation and function of FoxO1-mediated LOX-1+ M-MDSCs in relieving lupus-like symptoms
Source: Cell Death Dis. 2021 Sep 3;12(9):829. doi: 10.1038/s41419-021-04052-5 (PMC8417277; doi:10.1038/s41419-021-04052-5)

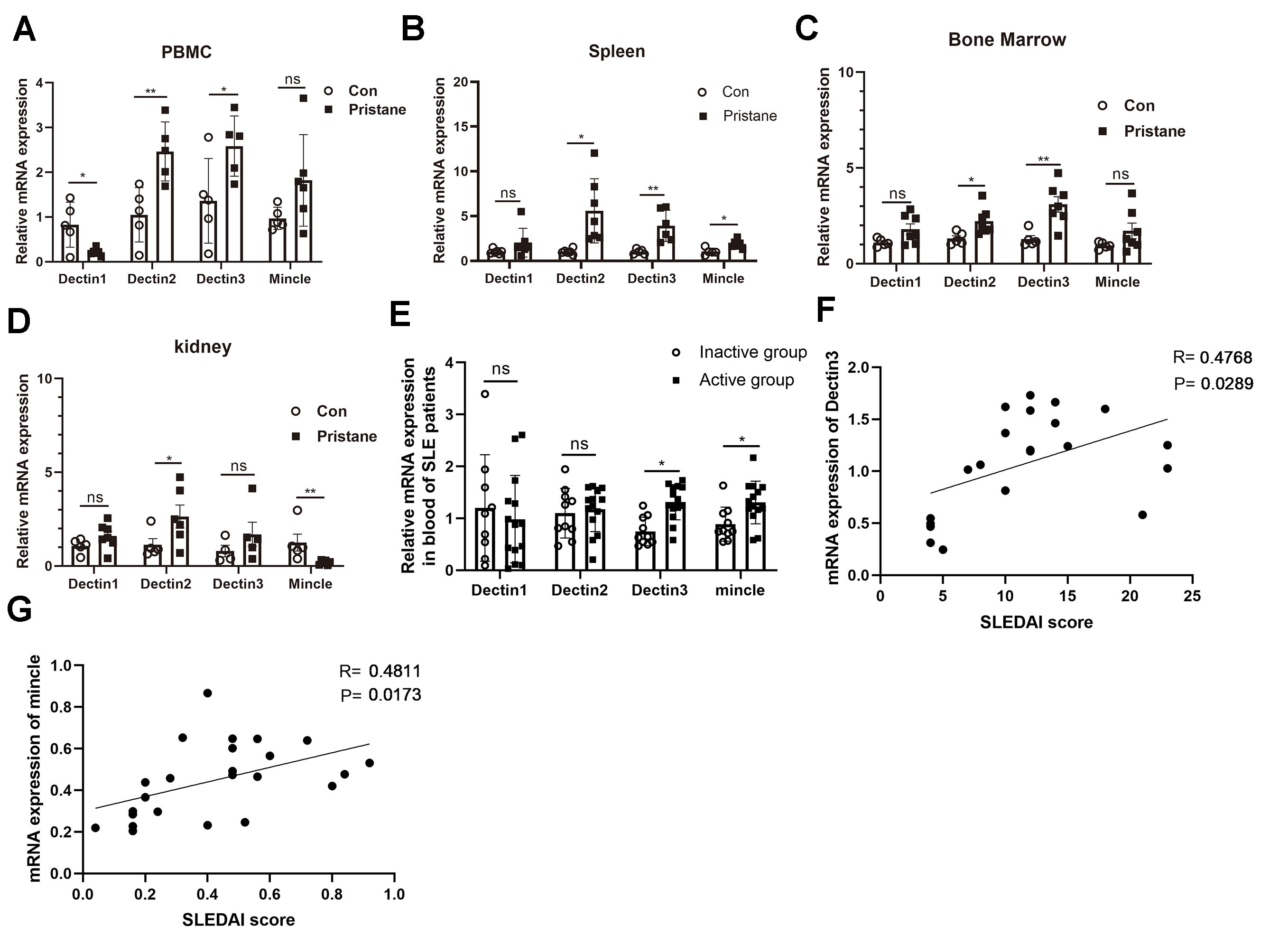

Supplement: Supplementary file 3 — Supplementary Figure.1 [file 41419_2021_4052_MOESM3_ESM.tif]

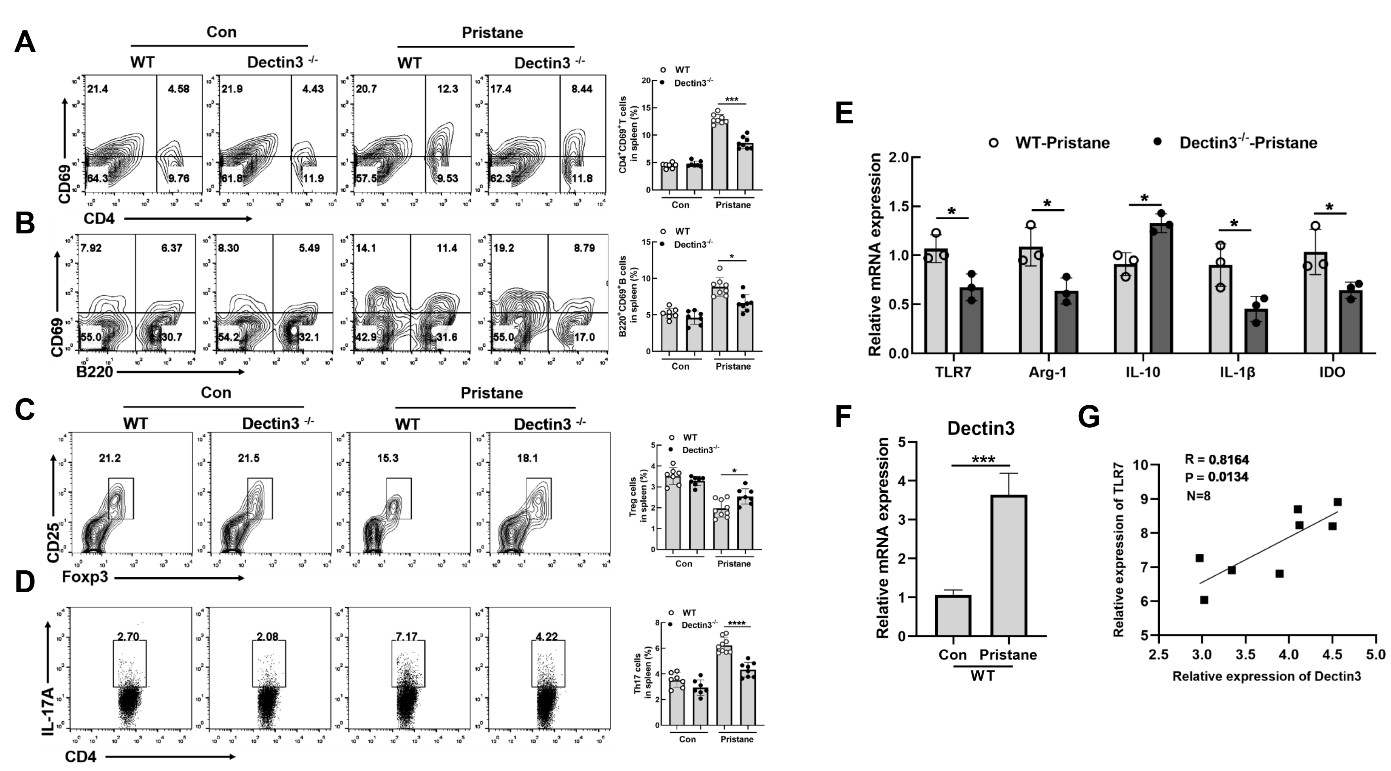

Supplement: Supplementary file 4 — Supplementary Figure.2 [file 41419_2021_4052_MOESM4_ESM.tif]

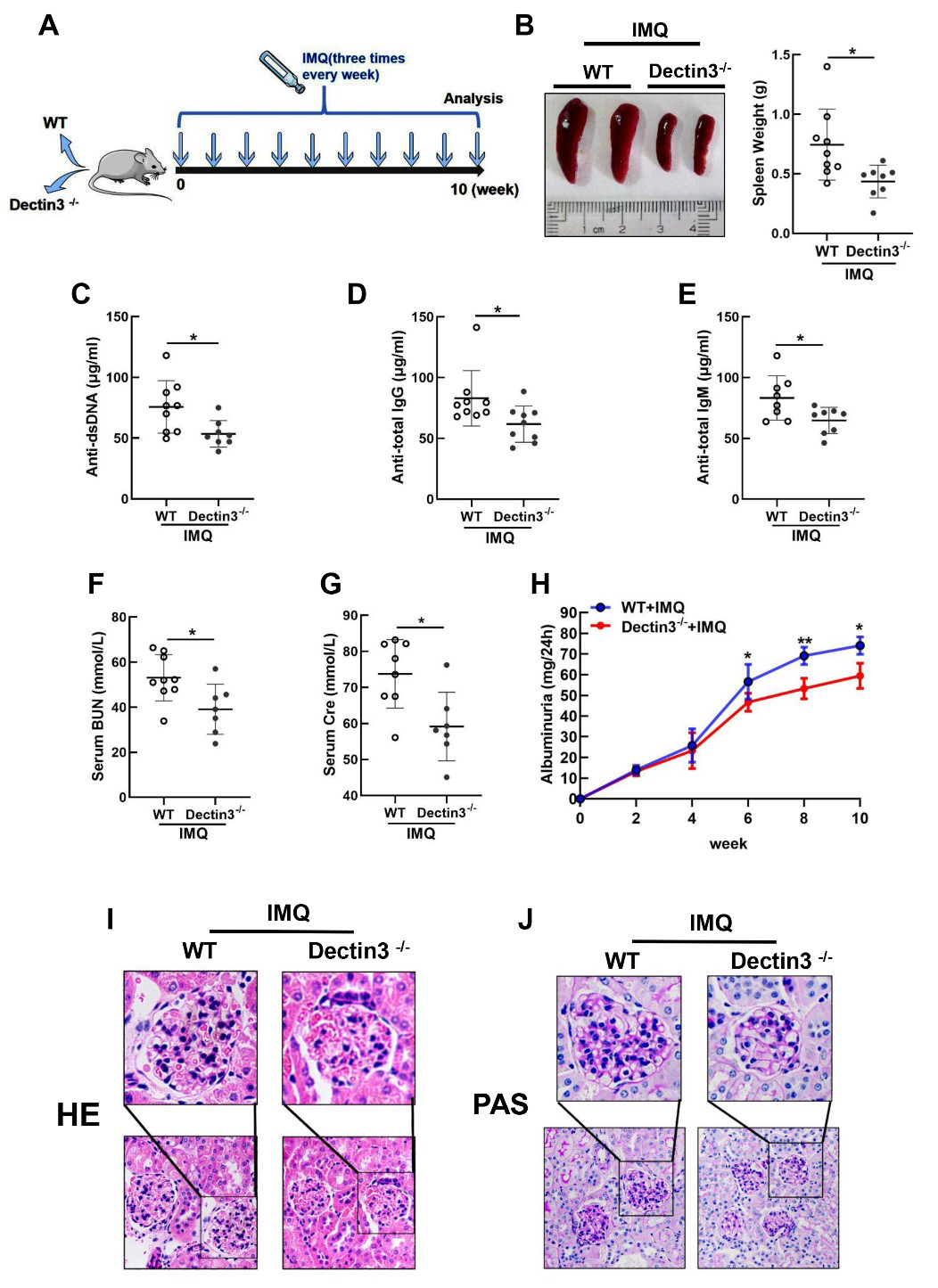

Supplement: Supplementary file 5 — Supplementary Figure.3 [file 41419_2021_4052_MOESM5_ESM.tif]

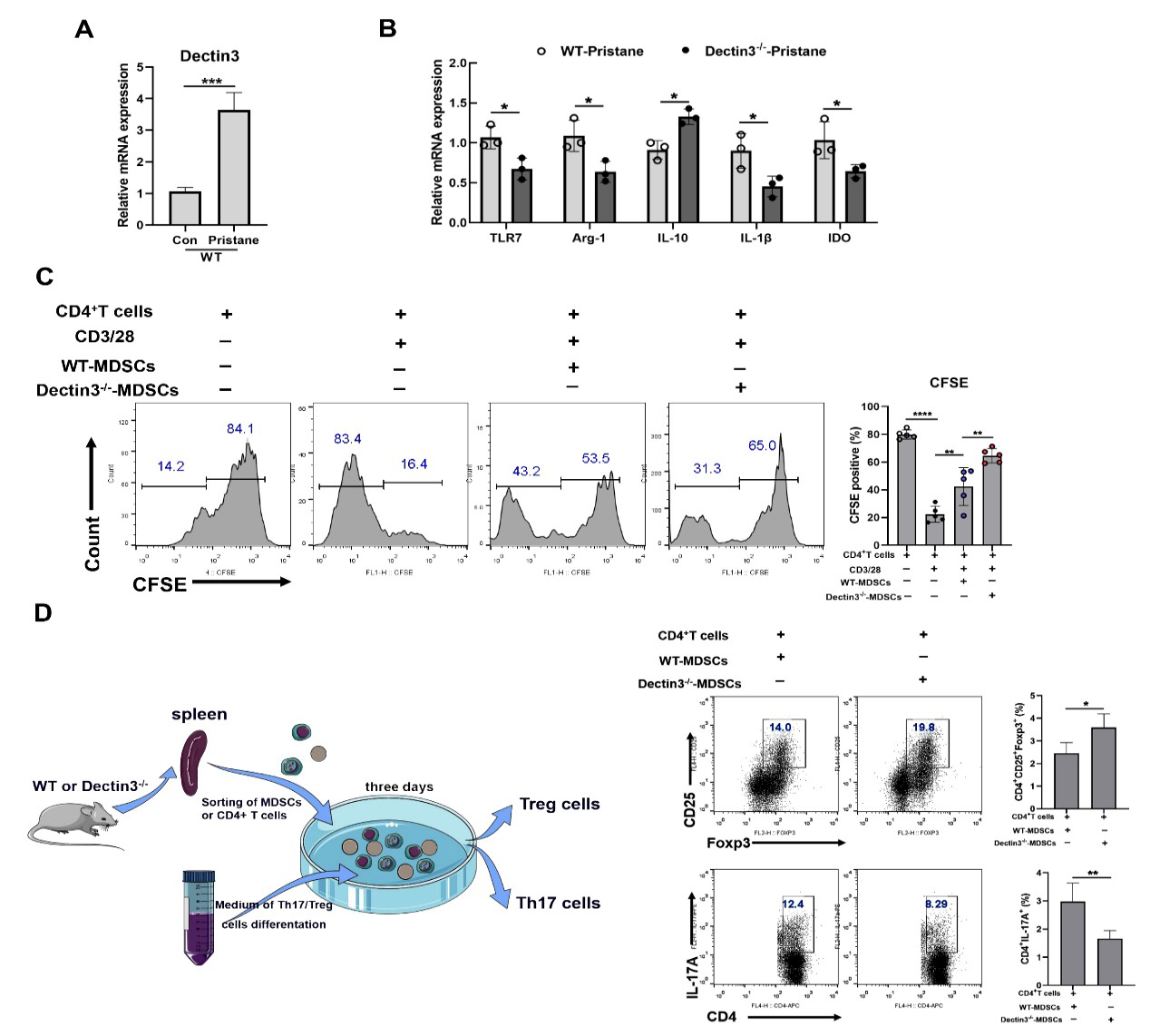

Supplement: Supplementary file 6 — Supplementary Figure.4 [file 41419_2021_4052_MOESM6_ESM.tif]

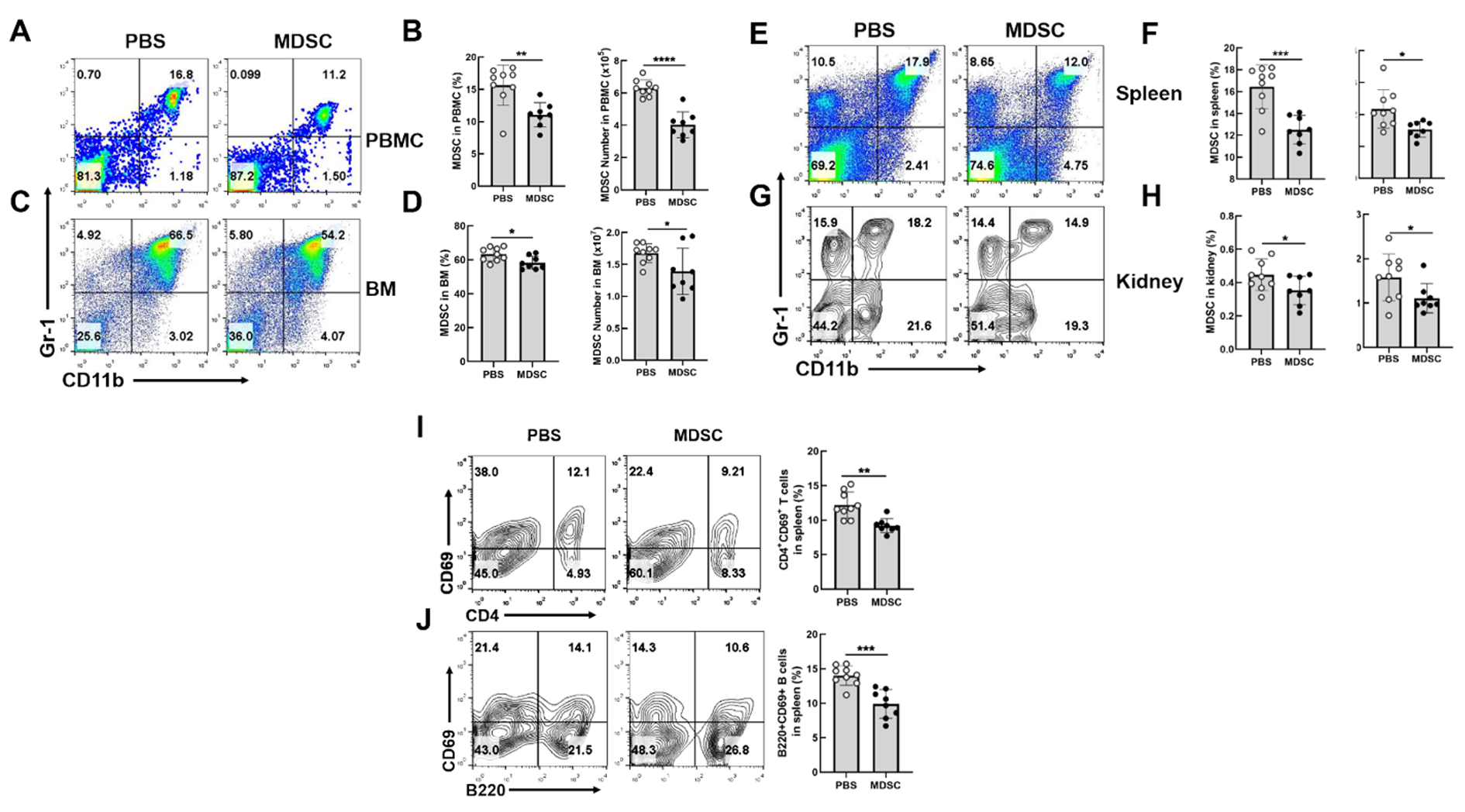

Supplement: Supplementary file 7 — Supplementary Figure.5 [file 41419_2021_4052_MOESM7_ESM.tif]

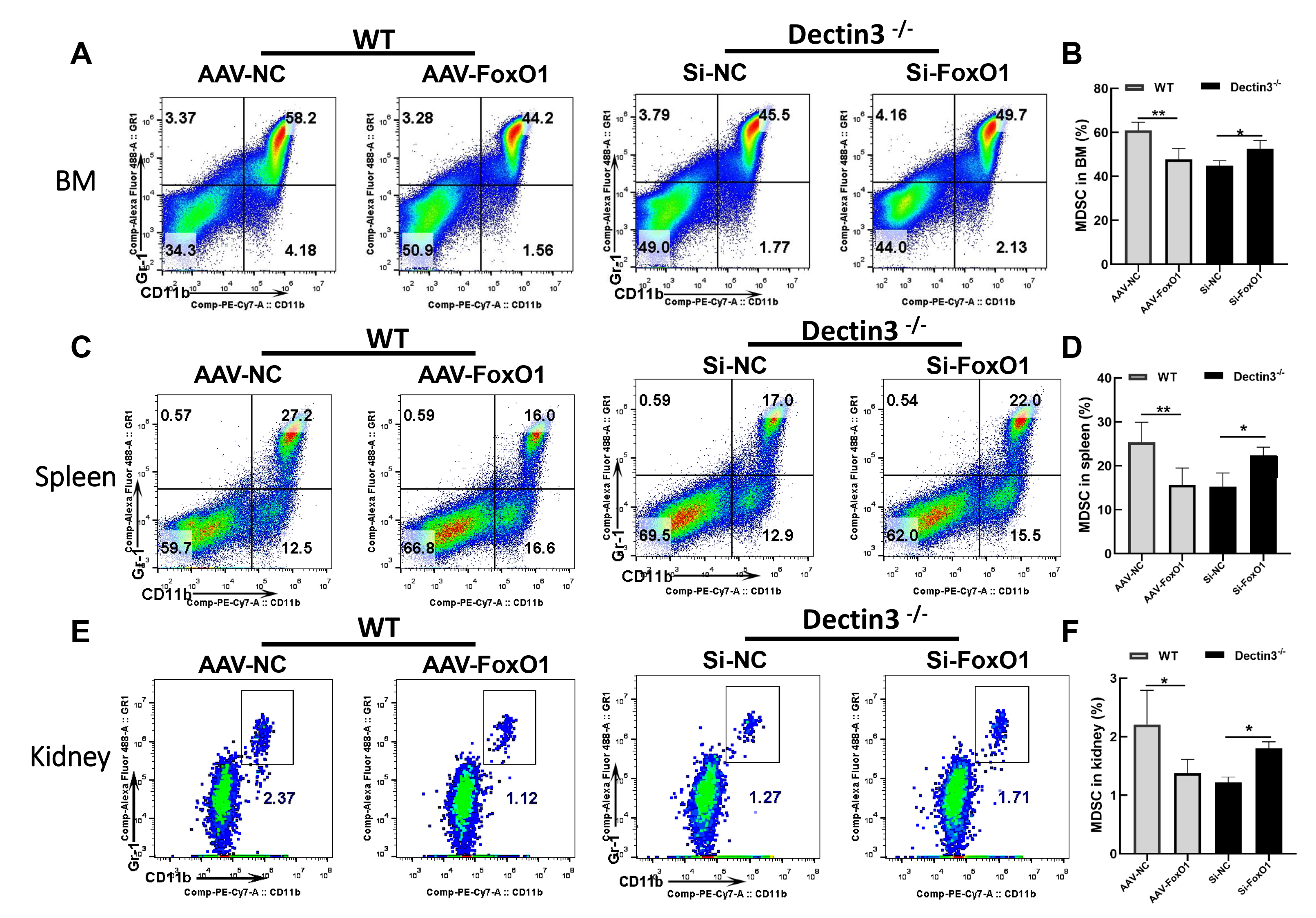

Supplement: Supplementary file 8 — Supplementary Figure.6 [file 41419_2021_4052_MOESM8_ESM.tif]

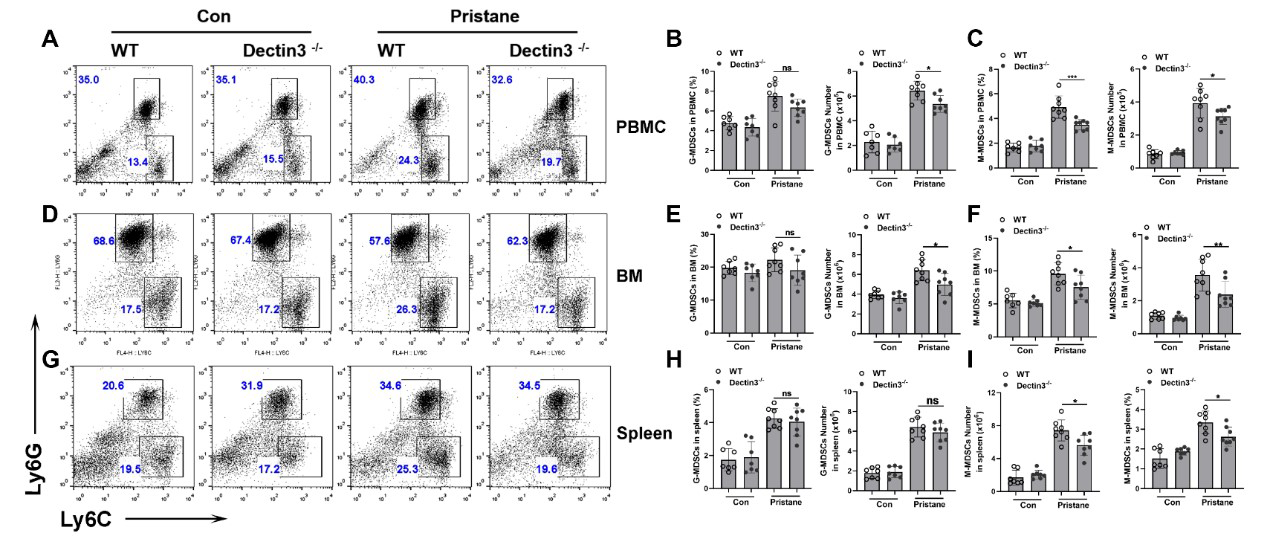

Supplement: Supplementary file 9 — Supplementary Figure.7 [file 41419_2021_4052_MOESM9_ESM.tif]

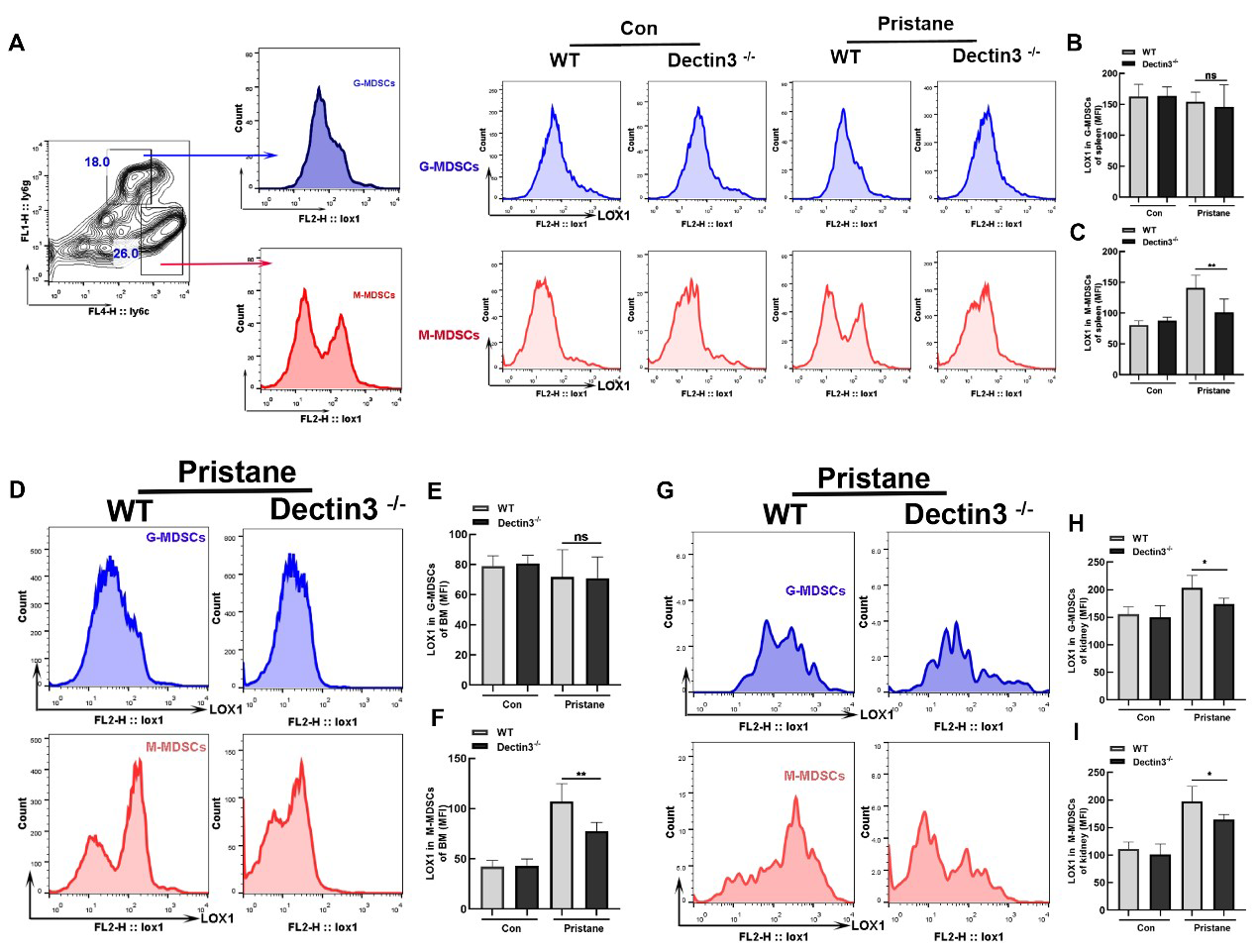

Supplement: Supplementary file 10 — Supplementary Figure.8 [file 41419_2021_4052_MOESM10_ESM.tif]
